# Supplementary material for: A protocol for a systematic review of process evaluations of interventions investigating sedentary behaviour in adults
Source: BMJ Open. 2019 Sep 18;9(9):e031291. doi: 10.1136/bmjopen-2019-031291 (PMC6756361; doi:10.1136/bmjopen-2019-031291)
Supplement: Supplementary data [file bmjopen-2019-031291supp002.pdf]

Additional file 2: Search strategies

CINHAL (EBSCOHost)

#	Query	Limiters/Expanders	Last Run Via	Results	Action
S40	S37 AND S38 AND S39	Search modes - Boolean/Phrase	Interface - EBSCOhost Research Databases Search Screen - Advanced Search Database - CINAHL	289	
S39	S33 OR S34 OR S35 or S36	Search modes - Boolean/Phrase	Interface - EBSCOhost Research Databases Search Screen - Advanced Search Database - CINAHL	56,168	
S38	S21 OR S22 OR S23 OR S24 OR S25 OR S26 OR S27 OR S28 OR S29 OR S30 OR S31	Search modes - Boolean/Phrase	Interface - EBSCOhost Research Databases Search Screen - Advanced Search Database - CINAHL	435,132	
S37	S1 OR S2 OR S3 OR S4 OR S5 OR S6 OR S7 OR S8 OR S9 OR S10 OR S11 OR S12 OR S13 OR S14 OR S15 OR S16 OR S17 OR S18 OR S19 OR S20	Search modes - Boolean/Phrase	Interface - EBSCOhost Research Databases Search Screen - Advanced Search Database - CINAHL	29,934	
S36	(MH "Process Assessment (Health Care)")	Search modes - Boolean/Phrase	Interface - EBSCOhost Research Databases Search Screen - Advanced Search Database - CINAHL	4,433	
S35	TX ((program* evaluat*))	Search modes - Boolean/Phrase	Interface - EBSCOhost Research Databases Search Screen - Advanced Search Database - CINAHL	52,056	

S34	Tx ((process* evaluat*))	Search modes - Boolean/Phrase	Interface - EBSCOhost Research Databases Search Screen - Advanced Search Database - CINAHL	1
S33	(MH "Program Evaluation")	Search modes - Boolean/Phrase	Interface - EBSCOhost Research Databases Search Screen - Advanced Search Database - CINAHL	34,068
S32	S21 or S22 or S23 or S24 or S25 or S26 or S27 or S28 or S29 or S30 or S31	Search modes - Boolean/Phrase	Interface - EBSCOhost Research Databases Search Screen - Advanced Search Database - CINAHL	435,132
S31	MH "Random Assignment"	Search modes - Boolean/Phrase	Interface - EBSCOhost Research Databases Search Screen - Advanced Search Database - CINAHL	53,614
S30	AB randomly	Search modes - Boolean/Phrase	Interface - EBSCOhost Research Databases Search Screen - Advanced Search Database - CINAHL	70,962
S29	AB randomized	Search modes - Boolean/Phrase	Interface - EBSCOhost Research Databases Search Screen - Advanced Search Database - CINAHL	129,654
S28	(MH "Clinical Trials+")	Search modes - Boolean/Phrase	Interface - EBSCOhost Research Databases Search Screen - Advanced Search Database - CINAHL	254,465
S27	PT Clinical trial	Search modes - Boolean/Phrase	Interface - EBSCOhost Research Databases Search Screen - Advanced Search Database - CINAHL	86,264

S26	TX clinic* n1 trial*	Search modes - Boolean/Phrase	Interface - EBSCOhost Research Databases Search Screen - Advanced Search Database - CINAHL	238,716
S25	TX randomi* control* trial*	Search modes - Boolean/Phrase	Interface - EBSCOhost Research Databases Search Screen - Advanced Search Database - CINAHL	164,354
S24	TX random* allocat*	Search modes - Boolean/Phrase	Interface - EBSCOhost Research Databases Search Screen - Advanced Search Database - CINAHL	9,911
S23	TX placebo*	Search modes - Boolean/Phrase	Interface - EBSCOhost Research Databases Search Screen - Advanced Search Database - CINAHL	55,417
S22	(MH "Placebos")	Search modes - Boolean/Phrase	Interface - EBSCOhost Research Databases Search Screen - Advanced Search Database - CINAHL	11,050
S21	TX allocat* random*	Search modes - Boolean/Phrase	Interface - EBSCOhost Research Databases Search Screen - Advanced Search Database - CINAHL	9,911
S20	TI (play* N5 ("video game*" or videogame* or "computer game*")) OR AB (play* N5 ("video game*" or videogame* or "computer game*"))	Search modes - Boolean/Phrase	Interface - EBSCOhost Research Databases Search Screen - Advanced Search Database - CINAHL	706
S19	TX ((watch* or view*) N5 (television	Search modes - Boolean/Phrase	Interface - EBSCOhost Research Databases Search Screen -	2,643

	or tv))		Advanced Search Database - CINAHL	
S18	TX (time N5 (computer* or television or tv or "video game*" or videogame* or gaming or screen or media))	Search modes - Boolean/Phrase	Interface - EBSCOhost Research Databases Search Screen - Advanced Search Database - CINAHL	4,338
S17	TX ((decrease or reduc* or discourag* or lessen*) N3 (sit or sitting or stand or standing or "physical* inactiv*"))	Search modes - Boolean/Phrase	Interface - EBSCOhost Research Databases Search Screen - Advanced Search Database - CINAHL	653
S16	TX ((light or low) N1 "physical activ*")	Search modes - Boolean/Phrase	Interface - EBSCOhost Research Databases Search Screen - Advanced Search Database - CINAHL	1,425
S15	TX "sit* less"	Search modes - Boolean/Phrase	Interface - EBSCOhost Research Databases Search Screen - Advanced Search Database - CINAHL	192
S14	TX "chair rise*"	Search modes - Boolean/Phrase	Interface - EBSCOhost Research Databases Search Screen - Advanced Search Database - CINAHL	234
S13	TI((computer* or television or tv or video game? or videogame? or gaming) and (sedentary or "physical* activity*" or sitting or seated or underactiv* or under activ*))	Search modes - Boolean/Phrase	Interface - EBSCOhost Research Databases Search Screen - Advanced Search Database - CINAHL	274

S12	TX (prolong* N2 (reclin* or sit or sitting or seated))	Search modes - Boolean/Phrase	Interface - EBSCOhost Research Databases Search Screen - Advanced Search Database - CINAHL	432
S11	TX ((sitting or lying) N2 posture*)	Search modes - Boolean/Phrase	Interface - EBSCOhost Research Databases Search Screen - Advanced Search Database - CINAHL	574
S10	TX "physical activity level*"	Search modes - Boolean/Phrase	Interface - EBSCOhost Research Databases Search Screen - Advanced Search Database - CINAHL	4,119
S9	TX (leisure time N5 ("physical* activ*" or passive or inactiv*)))	Search modes - Boolean/Phrase	Interface - EBSCOhost Research Databases Search Screen - Advanced Search Database - CINAHL	2,195
S8	TX "physical* inactiv*"	Search modes - Boolean/Phrase	Interface - EBSCOhost Research Databases Search Screen - Advanced Search Database - CINAHL	3,605
S7	TX "low energy expenditure"	Search modes - Boolean/Phrase	Interface - EBSCOhost Research Databases Search Screen - Advanced Search Database - CINAHL	55
S6	TX((inactiv* or no exercise or nonexercise or non exercise) N3 (adult* or men or women or males or females or individuals or people))	Search modes - Boolean/Phrase	Interface - EBSCOhost Research Databases Search Screen - Advanced Search Database - CINAHL	1,532
S5	TI (((sitting or sit or seated or stationary or standing) N3	Search modes - Boolean/Phrase	Interface - EBSCOhost Research Databases Search Screen -	2,598

	(task* or time or bout* or work* or break*))) OR AB (((sitting or sit or seated or stationary or standing) N3 (task* or time or bout* or work* or break*)))		Advanced Search Database - CINAHL	
S4	TX ((sedentary N3 (adult* or men or women or males or females or individuals or people or population*))	Search modes - Boolean/Phrase	Interface - EBSCOhost Research Databases Search Screen - Advanced Search Database - CINAHL	2,713
S3	TX ((sedentary or sitting or seated) N5 (behavio* or lifestyle or life-style)))	Search modes - Boolean/Phrase	Interface - EBSCOhost Research Databases Search Screen - Advanced Search Database - CINAHL	9,170
S2	TI (sedentary or sitting or sedentariness or sedentarism)	Search modes - Boolean/Phrase	Interface - EBSCOhost Research Databases Search Screen - Advanced Search Database - CINAHL	4,477
S1	(MH "Life Style, Sedentary")	Search modes - Boolean/Phrase	Interface - EBSCOhost Research Databases Search Screen - Advanced Search Database - CINAHL	6,680

SPORTDiscus (EBSCOHost)

	Query	Limiters/Expanders	Last Run Via	Results	Action
S39	S33 AND S37 AND S38	Search modes - Boolean/Phrase	Interface - EBSCOhost Research Databases Search Screen - Advanced Search Database - SPORTDiscus	37	
S38	S1 OR S2 OR S3 OR S4 OR S5 OR S6 OR S7 OR S8 OR S9 OR S10 OR S11 OR S12 OR S13 OR S14 OR S15 OR S16 OR S17 OR S18 OR S19 OR S20	Search modes - Boolean/Phrase	Interface - EBSCOhost Research Databases Search Screen - Advanced Search Database - SPORTDiscus	19,478	
S37	S34 OR S35 OR S36	Search modes - Boolean/Phrase	Interface - EBSCOhost Research Databases Search Screen - Advanced Search Database - SPORTDiscus	5,377	
S36	TI process* evaluat*	Search modes - Boolean/Phrase	Interface - EBSCOhost Research Databases Search Screen - Advanced Search Database - SPORTDiscus	295	
S35	TX program* evaluat*	Search modes - Boolean/Phrase	Interface - EBSCOhost Research Databases Search Screen - Advanced Search Database - SPORTDiscus	5,148	
S34	SU program evaluation	Search modes - Boolean/Phrase	Interface - EBSCOhost Research Databases Search Screen - Advanced Search Database - SPORTDiscus	1,209	
S33	S21 OR S22 OR S23 OR S24 OR S25 OR S26 OR S27 OR S28 OR S29 OR S30 OR	Search modes - Boolean/Phrase	Interface - EBSCOhost Research Databases Search Screen - Advanced Search	52,680	

	S31 OR S32		Database - SPORTDiscus	
S32	AB randomized	Search modes - Boolean/Phrase	Interface - EBSCOhost Research Databases Search Screen - Advanced Search Database - SPORTDiscus	19,923
S31	AB randomly	Search modes - Boolean/Phrase	Interface - EBSCOhost Research Databases Search Screen - Advanced Search Database - SPORTDiscus	15,478
S30	DE "CLINICAL trials"	Search modes - Boolean/Phrase	Interface - EBSCOhost Research Databases Search Screen - Advanced Search Database - SPORTDiscus	8,795
S29	TX clinic* n1 trial*	Search modes - Boolean/Phrase	Interface - EBSCOhost Research Databases Search Screen - Advanced Search Database - SPORTDiscus	15,367
S28	TX randomi* control* trial*	Search modes - Boolean/Phrase	Interface - EBSCOhost Research Databases Search Screen - Advanced Search Database - SPORTDiscus	17,436
S27	TX random* assign*	Search modes - Boolean/Phrase	Interface - EBSCOhost Research Databases Search Screen - Advanced Search Database - SPORTDiscus	8,031
S26	TX random* allocat*	Search modes - Boolean/Phrase	Interface - EBSCOhost Research Databases Search Screen - Advanced Search Database - SPORTDiscus	1,576
S25	TX placebo*	Search modes - Boolean/Phrase	Interface - EBSCOhost Research Databases Search Screen -	10,556

			Advanced Search Database - SPORTDiscus	
S24	DE "PLACEBOS (Medicine)"	Search modes - Boolean/Phrase	Interface - EBSCOhost Research Databases Search Screen - Advanced Search Database - SPORTDiscus	2,412
S23	DE "QUANTITATIVE research"	Search modes - Boolean/Phrase	Interface - EBSCOhost Research Databases Search Screen - Advanced Search Database - SPORTDiscus	1,448
S22	TX allocat* random*	Search modes - Boolean/Phrase	Interface - EBSCOhost Research Databases Search Screen - Advanced Search Database - SPORTDiscus	1,576
S21	((DE "RANDOMIZED controlled trials")))	Search modes - Boolean/Phrase	Interface - EBSCOhost Research Databases Search Screen - Advanced Search Database - SPORTDiscus	11,047
S20	TI (play* N5 ("video game*" or videogame* or "computer game*")) OR AB (play* N5 ("video game*" or videogame* or "computer game*"))	Search modes - Boolean/Phrase	Interface - EBSCOhost Research Databases Search Screen - Advanced Search Database - SPORTDiscus	564
S19	TI ((watch* or view*) N5 (television or tv)) OR AB ((watch* or view*) N5 (television or tv))	Search modes - Boolean/Phrase	Interface - EBSCOhost Research Databases Search Screen - Advanced Search Database - SPORTDiscus	2,377
S18	TI (time N5 (computer* or television or tv or "video game*" or videogame* or gaming or screen or	Search modes - Boolean/Phrase	Interface - EBSCOhost Research Databases Search Screen - Advanced Search Database - SPORTDiscus	1,711

	media)) OR AB (time N5 (computer* or television or tv or "video game*" or videogame* or gaming or screen or media))			
S17	TI ((decrease or reduc* or discourag* or lessen*) N3 (sit or sitting or stand or standing or "physical* inactiv*")) OR AB ((decrease or reduc* or discourag* or lessen*) N3 (sit or sitting or stand or standing or "physical* inactiv*"))	Search modes - Boolean/Phrase	Interface - EBSCOhost Research Databases Search Screen - Advanced Search Database - SPORTDiscus	303
S16	TI ((light or low) N1 "physical activ*") OR AB ((light or low) N1 "physical activ*")	Search modes - Boolean/Phrase	Interface - EBSCOhost Research Databases Search Screen - Advanced Search Database - SPORTDiscus	614
S15	TI "sit* less" OR AB "sit* less"	Search modes - Boolean/Phrase	Interface - EBSCOhost Research Databases Search Screen - Advanced Search Database - SPORTDiscus	41
S14	TI "chair rise*" OR AB "chair rise*"	Search modes - Boolean/Phrase	Interface - EBSCOhost Research Databases Search Screen - Advanced Search Database - SPORTDiscus	75
S13	TX((computer* or television or tv or video game? or videogame? or gaming) and (sedentary or physical* activity* or sitting or seated or underactiv* or under	Search modes - Boolean/Phrase	Interface - EBSCOhost Research Databases Search Screen - Advanced Search Database - SPORTDiscus	2,498

	activ*))			
S12	TI (prolong* N2 (reclin* or sit or sitting or seated)) OR AB (prolong* N2 (reclin* or sit or sitting or seated))	Search modes - Boolean/Phrase	Interface - EBSCOhost Research Databases Search Screen - Advanced Search Database - SPORTDiscus	203
S11	TI ((sitting or lying) N2 posture*) OR AB ((sitting or lying) N2 posture*)	Search modes - Boolean/Phrase	Interface - EBSCOhost Research Databases Search Screen - Advanced Search Database - SPORTDiscus	321
S10	TI "physical activity level*" OR AB "physical activity level*"	Search modes - Boolean/Phrase	Interface - EBSCOhost Research Databases Search Screen - Advanced Search Database - SPORTDiscus	2,795
S9	TI (leisure time N5 ("physical* activ*" or passive or inactiv*))) OR AB (leisure time N5 ("physical* activ*" or passive or inactiv*))	Search modes - Boolean/Phrase	Interface - EBSCOhost Research Databases Search Screen - Advanced Search Database - SPORTDiscus	1,268
S8	TI "physical* inactiv*" OR AB "physical* inactiv*"	Search modes - Boolean/Phrase	Interface - EBSCOhost Research Databases Search Screen - Advanced Search Database - SPORTDiscus	1,626
S7	TI "low energy expenditure" OR AB "low energy expenditure"	Search modes - Boolean/Phrase	Interface - EBSCOhost Research Databases Search Screen - Advanced Search Database - SPORTDiscus	25
S6	TX(inactiv* or no exercise or nonexercise or non exercise) N3 (adult* or men or women or males or females or individuals or	Search modes - Boolean/Phrase	Interface - EBSCOhost Research Databases Search Screen - Advanced Search Database - SPORTDiscus	1,073

	people))			
S5	TI (((sitting or sit or seated or stationary or standing) N3 (task* or time or bout* or work* or break*))) OR AB (((sitting or sit or seated or stationary or standing) N3 (task* or time or bout* or work* or break*)))	Search modes - Boolean/Phrase	Interface - EBSCOhost Research Databases Search Screen - Advanced Search Database - SPORTDiscus	1,733
S4	TI ((sedentary N3 (adult* or men or women or males or females or individuals or people or population*))) OR AB ((sedentary N3 (adult* or men or women or males or females or individuals or people or population*)))	Search modes - Boolean/Phrase	Interface - EBSCOhost Research Databases Search Screen - Advanced Search Database - SPORTDiscus	2,922
S3	TI ((sedentary or sitting or seated) N5 (behavio* or lifestyle or life-style))) OR AB (seated) N5 (behavio* or lifestyle or life-style)) OR AB ((sedentary or sitting or seated) N5 (behavio* or lifestyle or life-style))) OR AB (seated) N5 (behavio* or lifestyle or life-style))	Search modes - Boolean/Phrase	Interface - EBSCOhost Research Databases Search Screen - Advanced Search Database - SPORTDiscus	2,419
S2	TI (sedentary or sitting or sedentariness or sedentarism)	Search modes - Boolean/Phrase	Interface - EBSCOhost Research Databases Search Screen - Advanced Search Database - SPORTDiscus	3,110

S1	SU Sedentary Lifestyle	Search modes - Boolean/Phrase	Interface - EBSCOhost Research Databases<br>Search Screen - Advanced Search<br>Database - SPORTDiscus	

Cochrane Database of Systematic Reviews (Wiley)

#1 MeSH descriptor: [Sedentary Behavior] this term only 906

#2 sedentary or sitting or sedentariness or sedentarism:ti 9230

#3 (sedentary or sitting or seated) near/5 (behavio* or lifestyle or life-style):ti,ab,kw (Word variations have been searched) 2297

#4 sedentary near/3 (adult* or men or women or males or females or individuals or people or population*):ti,ab,kw (Word variations have been searched) 1920

#5 (sitting or sit or seated or stationary or standing) near/3 (task* or time or bout* or work* or break*):ti,ab,kw (Word variations have been searched) 1265

#6 ((inactiv* or no exercise or nonexercise or non exercise) near/3 (adult* or men or women or males or females or individuals or people)):ti,ab,kw 13841

#7 "low energy expenditure":ti,ab,kw (Word variations have been searched) 10

#8 "physical* inactiv*":ti,ab,kw (Word variations have been searched) 0

#9 "leisure time" near/5 ("physical* activ*" or passive or inactiv*):ti,ab,kw (Word variations have been searched) 224

#10 "physical activity level*":ti,ab,kw (Word variations have been searched) 1459

#11 (sitting or lying) near/2 posture*:ti,ab,kw (Word variations have been searched) 138

#12 prolong* near/2 (reclin* or sit or sitting or seated):ti,ab,kw (Word variations have been searched) 183

#13 "chair rise*":ti,ab,kw (Word variations have been searched) 178

#14 "sit* less":ti,ab,kw (Word variations have been searched) 30

#15 (light or low) near/1 "physical activ*":ti,ab,kw (Word variations have been searched) 226

#16 time near/5 (computer* or television or tv or "video game*" or videogame* or gaming or screen or media):ti,ab,kw (Word variations have been searched) 2056

#17 (watch* or view*) near/5 (television or tv):ti,ab,kw (Word variations have been searched) 464

#18 play* near/5 ("video game*" or videogame* or "computer game*"):ti,ab,kw (Word variations have been searched) 291

#19 (decrease or reduc* or discourag* or lessen*) near/3 (sit or sitting or stand or standing or "physical* inactiv*"):ti,ab,kw (Word variations have been searched) 751

#20 ((computer* or television or tv or video game* or videogame* or gaming) and (sedentary or physical* activity* or sitting or seated or underactiv* or under activ*)):ti 124

#21 {or #1-#20} 27029

#22 MeSH descriptor: [Program Evaluation] this term only 5548

#23 "program* evaluation*":ti,ab,kw 6047

#24 "process* evaluation*":ti,ab,kw 1318

#25 MeSH descriptor: [Process Assessment (Health Care)] this term only 193

#26 {or #22-#25} 7251

#27 #21 and #26 483

Cochrane Central Register of Controlled Trials (Wiley)

#1 MeSH descriptor: [Sedentary Behavior] this term only 906

#2 sedentary or sitting or sedentariness or sedentarism:ti 9230

#3 (sedentary or sitting or seated) near/5 (behavio* or lifestyle or life-style):ti,ab,kw (Word variations have been searched) 2297

#4 sedentary near/3 (adult* or men or women or males or females or individuals or people or population*):ti,ab,kw (Word variations have been searched) 1920

#5 (sitting or sit or seated or stationary or standing) near/3 (task* or time or bout* or work* or break*):ti,ab,kw (Word variations have been searched) 1265

#6 ((inactiv* or no exercise or nonexercise or non exercise) near/3 (adult* or men or women or males or females or individuals or people)):ti,ab,kw 13841

#7 "low energy expenditure":ti,ab,kw (Word variations have been searched) 10

#8 ("physical* inactive" or "physical inactivity"):ti,ab,kw (Word variations have been searched) 987

#9 "leisure time" near/5 ("physical* activ*" or passive or inactiv*):ti,ab,kw (Word variations have been searched) 224

#10 "physical activity level*":ti,ab,kw (Word variations have been searched) 1459

#11 (sitting or lying) near/2 posture*:ti,ab,kw (Word variations have been searched) 138

#12 prolong* near/2 (reclin* or sit or sitting or seated):ti,ab,kw (Word variations have been searched) 183

#13 "chair rise*":ti,ab,kw (Word variations have been searched) 178

#14 "sit* less":ti,ab,kw (Word variations have been searched) 30

#15 (light or low) near/1 "physical activ*":ti,ab,kw (Word variations have been searched) 226

#16 time near/5 (computer* or television or tv or "video game*" or videogame* or gaming or screen or media):ti,ab,kw (Word variations have been searched) 2056

#17 (watch* or view*) near/5 (television or tv):ti,ab,kw (Word variations have been searched) 464

#18 play* near/5 ("video game*" or videogame* or "computer game*"):ti,ab,kw (Word variations have been searched) 291

#19 (decrease or reduc* or discourag* or lessen*) near/3 (sit or sitting or stand or standing or "physical* inactiv*"):ti,ab,kw (Word variations have been searched) 751

#20 ((computer* or television or tv or video game* or videogame* or gaming) and (sedentary or physical* activity* or sitting or seated or underactiv* or under activ*)):ti 124

#21 {or #1-#20} 27534

#22 MeSH descriptor: [Program Evaluation] this term only 5548

#23 ("program* evaluation*"):ti,ab,kw6047

#24 "process* evaluation*":ti,ab,kw 1318

#25 MeSH descriptor: [Process Assessment (Health Care)] this term only 193

#26 {or #22-#25} 7251

#27 #21 and #26 in Trials 486

AMED (Allied and Complementary Medicine)

1 Sedentary Lifestyle/ (292)

2 (sedentary or sitting or sedentariness or sedentarism).ti. (702)

3 ((sedentary or sitting or seated) adj5 (behavio* or lifestyle or life-style)).tw. (471)

4 ((inactiv* or no exercise or nonexercise or non exercise) adj3 (adult? or men or women or males or females or individuals or people)).tw. (1509)

5 (sedentary adj3 (adult? or men or women or males or females or individuals or people or population?)).tw. (382)

6 ((sitting or sit or seated or stationary or standing) adj3 (task* or time or bout* or work* or break*)).tw. (563)

7 low energy expenditure.tw. (4)

8 physical* inactiv*.tw. (216)

9 (leisure time adj5 (physical* activ* or passive or inactiv*)).tw. (152)

10 "physical activity level*".tw. (405)

11 ((sitting or lying) adj2 posture*).tw. (210)

12 (prolong* adj2 (reclin* or sit or sitting or seated)).tw. (56)

13 chair rise?.tw. (58)

14 "sit* less".tw. (13)

15 ((light or low) adj "physical activ*").tw. (48)

16 ((decrease or reduc* or discourag* or lessen*) adj3 (sit or sitting or stand or standing or physical* inactiv*)).tw. (95)

17 (time adj5 (computer* or television or tv or video game? or videogame? or gaming or screen or media)).tw. (189)

18 ((watch* or view*) adj5 (television or tv)).tw. (69)

19 (play* adj5 (video game? or videogame? or computer game?)).tw. (47)

20 ((computer* or television or tv or video game? or videogame? or gaming) and (sedentary or physical* activity* or sitting or seated or underactiv* or under activ*)).ti. (99)

21 or/1-20 [sedentary behaviour terms] (4197)

22 process evaluat*.mp. (88)

23 "Outcome and Process Assessment"/ (1147)

24 program evaluat*.mp. (2347)

25 or/22-24 [process evaluation] (3523)

26 21 and 25 [sedentary behaviour and process evaluation] (76)

EMBASE (OVID)

1 Sedentary Lifestyle/ (11663)

2 (sedentary or sitting or sedentariness or sedentarism).ti. (7958)

3 ((sedentary or sitting or seated) adj5 (behavio* or lifestyle or life-style)).tw. (11608)

4 ((inactiv* or no exercise or nonexercise or non exercise) adj3 (adult? or men or women or males or females or individuals or people)).tw. (3191)

5 (sedentary adj3 (adult? or men or women or males or females or individuals or people or population?)).tw. (6088)

6 ((sitting or sit or seated or stationary or standing) adj3 (task* or time or bout* or work* or break*)).tw. (6389)

7 low energy expenditure.tw. (196)

8 physical* inactiv*.tw. (10206)

9 (leisure time adj5 (physical* activ* or passive or inactiv*)).tw. (4182)

10 "physical activity level*".tw. (10320)

11 ((sitting or lying) adj2 posture*).tw. (1181)

12 (prolong* adj2 (reclin* or sit or sitting or seated)).tw. (820)

13 chair rise?.tw. (561)

14 "sit* less".tw. (1080)

15 ((light or low) adj "physical activ*").tw. (3123)

16 ((decrease or reduc* or discourag* or lessen*) adj3 (sit or sitting or stand or standing or physical* inactiv*)).tw. (1570)

17 (time adj5 (computer* or television or tv or video game? or videogame? or gaming or screen or media)).tw. (12058)

18 ((watch* or view*) adj5 (television or tv)).tw. (5775)

19 (play* adj5 (video game? or videogame? or computer game?)).tw. (1941)

20 ((computer* or television or tv or video game? or videogame? or gaming) and (sedentary or physical* activity* or sitting or seated or underactiv* or under activ*)).ti. (432)

21 or/1-20 [sedentary behaviour terms] (72983)

22 Randomized controlled trial/ (489967)

23 Controlled clinical study/ (413369)

24 22 or 23 (647900)

25 Random*.tw. (1243699)

26 randomization/ (72965)

27 intermethod comparison/ (235116)

28 placebo.tw. (233779)

29 (compare or compared or comparison).ti. (353942)

30 ((evaluated or evaluate or evaluating or assessed or assess) and (compare or compared or comparing or comparison)).ab. (1742773)

31 (open adj label).tw. (67575)

32 ((double or single or doubly or singly) adj (blind or blinded or blindly)).tw. (166412)

33 double blind procedure/ (131359)

34 parallel group*1.tw. (20690)

35 (crossover or cross over).tw. (76213)

36 ((assign* or match or matched or allocation) adj5 (alternate or group*1 or intervention*1 or patient*1 or subject*1 or participant*1)).tw. (265294)

37 (assigned or allocated).tw. (310200)

38 (controlled adj7 (study or design or trial)).tw. (280066)

39 (volunteer or volunteers).tw. (180358)

40 human experiment/ (307840)

41 trial.ti. (231364)

42 or/25-41 (3776672)

43 42 and 24 (520100)

44 (random* adj sampl* adj7 ("cross section*" or questionnaire*1 or survey* or database*1)).tw. not (comparative study/ or controlled study/ or randomi?ed controlled.tw. or randomly assigned.tw.) (6701)

45 Cross-sectional study/ not (randomized controlled trial/ or controlled clinical study/ or controlled study/ or randomi?ed controlled.tw. or control group*1.tw.) (196889)

46 (((case adj control*) and random*) not randomi?ed controlled).tw. (14459)

47 (Systematic review not (trial or study)).ti. (111407)

48 (nonrandom* not random*).tw. (12428)

49 "Random field*".tw. (1913)

50 (random cluster adj3 sampl*).tw. (1109)

51 (review.ab. and review.pt.) not trial.ti. (671976)

52 "we searched".ab. and (review.ti. or review.pt.) (26759)

53 "update review".ab. (89)

54 (databases adj4 searched).ab. (27931)

55 (rat or rats or mouse or mice or swine or porcine or murine or sheep or lambs or pigs or piglets or rabbit or rabbits or cat or cats or dog or dogs or cattle or bovine or monkey or monkeys or trout or marmoset*1).ti. and animal experiment/ (621221)

56 Animal experiment/ not (human experiment/ or human/) (1278682)

57 or/44-56 (2292431)

58 43 not 57 [Cochrane Embase RTC search filter Jan 2015] (504463)

59 program evaluat*.mp. (15961)

60 health care quality/ (199019)

61 process* evaluat*.mp. (3994)

62 or/59-61 [process evaluation] (216384)

63 21 and 58 and 62 [sedentary behaviour and RCTs and process evaluations] (213)

64 remove duplicates from 63 (209)

PsycINFO (OVID)

1 SEDENTARY BEHAVIOR/ (45)

2 (sedentary or sitting or sedentariness or sedentarism).ti. (7958)

3 ((sedentary or sitting or seated) adj5 (behavio* or lifestyle or life-style)).tw. (11608)

4 ((inactiv* or no exercise or nonexercise or non exercise) adj3 (adult? or men or women or males or females or individuals or people)).tw. (3191)

5 (sedentary adj3 (adult? or men or women or males or females or individuals or people or population?)).tw. (6088)

6 ((sitting or sit or seated or stationary or standing) adj3 (task* or time or bout* or work* or break*)).tw. (6389)

7 low energy expenditure.tw. (196)

8 physical* inactiv*.tw. (10206)

9 (leisure time adj5 (physical* activ* or passive or inactiv*)).tw. (4182)

10 "physical activity level*".tw. (10320)

11 ((sitting or lying) adj2 posture*).tw. (1181)

12 (prolong* adj2 (reclin* or sit or sitting or seated)).tw. (820)

13 chair rise?.tw. (561)

14 "sit* less".tw. (1080)

15 ((light or low) adj "physical activ*").tw. (3123)

16 ((decrease or reduc* or discourag* or lessen*) adj3 (sit or sitting or stand or standing or physical* inactiv*)).tw. (1570)

17 (time adj5 (computer* or television or tv or video game? or videogame? or gaming or screen or media)).tw. (12058)

18 ((watch* or view*) adj5 (television or tv)).tw. (5775)

19 (play* adj5 (video game? or videogame? or computer game?)).tw. (1941)

20 ((computer* or television or tv or video game? or videogame? or gaming) and (sedentary or physical* activity* or sitting or seated or underactiv* or under activ*)).ti. (432)

21 or/1-20 [sedentary behaivour] (68730)

22 Treatment Effectiveness Evaluation/ (0)

23 exp Treatment Outcomes/ (0)

24 Psychotherapeutic Outcomes/ (0)

25 PLACEBO/ (277088)

26 exp Followup Studies/ (0)

27 placebo*.tw. (235252)

28 random*.tw. (1243699)

29 comparative stud*.tw. (77003)

30 (clinical adj3 trial*).tw. (433925)

31 (research adj3 design).tw. (31590)

32 (evaluat* adj3 stud*).tw. (605716)

33 (prospectiv* adj3 stud*).tw. (453513)

34 ((singl* or doubl*or trebl* or tripl*) adj3 (blind* or mask*)).tw. (24389)

35 or/22-34 [RCT filter adapted from Watson RJ, Richardson PH 1999] (2613798)

36 program evaluat*.mp. (15961)

37 process* evaluat*.mp. (3994)

38 evaluation/ (130069)

39 or/36-38 [process evaluation terms] (148923)

40 21 and 35 and 39 [sedentary behaviour and rcts and process evalutions] (267)

Ovid MEDLINE(R)

1 Sedentary Lifestyle/ (7525)

2 (sedentary or sitting or sedentariness or sedentarism).ti. (6452)

3 ((sedentary or sitting or seated) adj5 (behavio* or lifestyle or life-style)).tw. (7249)

4 ((inactiv* or no exercise or nonexercise or non exercise) adj3 (adult? or men or women or males or females or individuals or people)).tw. (2515)

5 (sedentary adj3 (adult? or men or women or males or females or individuals or people or population?)).tw. (4859)

6 ((sitting or sit or seated or stationary or standing) adj3 (task* or time or bout* or work* or break*)).tw. (4603)

7 low energy expenditure.tw. (144)

8 physical* inactiv*.tw. (6591)

9 (leisure time adj5 (physical* activ* or passive or inactiv*)).tw. (3445)

10 "physical activity level*".tw. (6404)

11 ((sitting or lying) adj2 posture*).tw. (998)

12 (prolong* adj2 (reclin* or sit or sitting or seated)).tw. (564)

13 chair rise?.tw. (323)

14 "sit* less".tw. (601)

15 ((light or low) adj "physical activ*").tw. (1853)

16 ((decrease or reduc* or discourag* or lessen*) adj3 (sit or sitting or stand or standing or physical* inactiv*)).tw. (1377)

17 (time adj5 (computer* or television or tv or video game? or videogame? or gaming or screen or media)).tw. (8936)

18 ((watch* or view*) adj5 (television or tv)).tw. (4240)

19 (play* adj5 (video game? or videogame? or computer game?)).tw. (1305)

20 ((computer* or television or tv or video game? or videogame? or gaming) and (sedentary or physical* activity* or sitting or seated or underactiv* or under activ*)).ti. (351)

21 or/1-20 [sedentary behaviour terms] (50991)

22 Program Evaluat*.mp. (62861)

23 "Outcome and Process Assessment (Health Care)"/ (25572)

24 "Process Assessment (Health Care)"/ (4358)

25 process evaluat*.mp. (2608)

26 or/22-25 [process evaluation] (91311)

27 randomized controlled trial.pt. (476630)

28 controlled clinical trial.pt. (92914)

29 randomized.ab. (377791)

30 placebo.ab. (177752)

31 drug therapy.fs. (2086845)

32 randomly.ab. (262246)

33 trial.ab. (392148)

34 groups.ab. (1631334)

35 27 or 28 or 29 or 30 or 31 or 32 or 33 or 34 (4041965)

36 exp animals/ not humans.sh. (4552221)

37 35 not 36 [Cochrane RCT filter 2008, sensitivity maximimising] (3448772)

38 21 and 26 and 37 [sedentary behaviour and process evaluation and RCTs] (420)

OVID MEDLINE(R) and Epub Ahead of Print, In-Process & Other Non-Indexed Citations

1 Sedentary Lifestyle/ (7594)

2 (sedentary or sitting or sedentariness or sedentarism).ti. (7865)

3 ((sedentary or sitting or seated) adj5 (behavio* or lifestyle or life-style)).tw. (8993)

4 ((inactiv* or no exercise or nonexercise or non exercise) adj3 (adult? or men or women or males or females or individuals or people)).tw. (2927)

5 (sedentary adj3 (adult? or men or women or males or females or individuals or people or population?)).tw. (5551)

6 ((sitting or sit or seated or stationary or standing) adj3 (task* or time or bout* or work* or break*)).tw. (5742)

7 low energy expenditure.tw. (166)

8 physical* inactiv*.tw. (7962)

9 (leisure time adj5 (physical* activ* or passive or inactiv*)).tw. (3859)

10 "physical activity level*".tw. (7765)

11 ((sitting or lying) adj2 posture*).tw. (1165)

12 (prolong* adj2 (reclin* or sit or sitting or seated)).tw. (721)

13 chair rise?.tw. (374)

14 "sit* less".tw. (693)

15 ((light or low) adj "physical activ*").tw. (2305)

16 ((decrease or reduc* or discourag* or lessen*) adj3 (sit or sitting or stand or standing or physical* inactiv*)).tw. (1670)

17 (time adj5 (computer* or television or tv or video game? or videogame? or gaming or screen or media)).tw. (10911)

18 ((watch* or view*) adj5 (television or tv)).tw. (4851)

19 (play* adj5 (video game? or videogame? or computer game?)).tw. (1650)

20 ((computer* or television or tv or video game? or videogame? or gaming) and (sedentary or physical* activity* or sitting or seated or underactiv* or under activ*)).ti. (415)

21 or/1-20 [sedentary behaviour terms] (60730)

22 Program Evaluat*.mp. (62861)

23 "Outcome and Process Assessment (Health Care)"/ (25610)

24 "Process Assessment (Health Care)"/ (4370)

25 process evaluat*.mp. (3311)

26 or/22-25 [process evaluation] (93381)

27 randomized controlled trial.pt. (477874)

28 controlled clinical trial.pt. (92968)

29 randomized.ab. (437254)

30 placebo.ab. (196103)

31 drug therapy.fs. (2090621)

32 randomly.ab. (307192)

33 trial.ab. (456911)

34 groups.ab. (1890503)

35 27 or 28 or 29 or 30 or 31 or 32 or 33 or 34 (4396098)

36 exp animals/ not humans.sh. (4557181)

37 35 not 36 [Cochrane RCT filter 2008, sensitivity maximimising] (3801789)

38 21 and 26 and 37 [sedentary behaviour and process evaluation and RCTs] (449)

Web of Science (Clarivate)

Set	Results	Save History / Create Alert · Open Saved History	Sets
# 23	121	#22 AND #21 AND #18<br>*Indexes=SCI-EXPANDED, SSCI, CPCI-S, CPCI-SSH Timespan=1900-2019*	Edit
# 22	149,472	#1 or #2 or #3 or #4 or #5 or #6 or #7 or #8 or #9 or #10 or #11 or #12 or #13 or #14 or #15 or #16 or #17<br>*Indexes=SCI-EXPANDED, SSCI, CPCI-S, CPCI-SSH Timespan=1900-2019*	Edit
# 21	13,136	#20 OR #19<br>*Indexes=SCI-EXPANDED, SSCI, CPCI-S, CPCI-SSH Timespan=1900-2019*	Edit
# 20	4,451	TS=("process evaluat*")<br>*Indexes=SCI-EXPANDED, SSCI, CPCI-S, CPCI-SSH Timespan=1900-2019*	Edit
# 19	8,811	TS=("program* evaluat*")<br>*Indexes=SCI-EXPANDED, SSCI, CPCI-S, CPCI-SSH Timespan=1900-2019*	Edit
# 18	2,014,426	TOPIC: (random* or RCT or placebo or clinical Near/1 trial*)<br>*Indexes=SCI-EXPANDED, SSCI, CPCI-S, CPCI-SSH Timespan=1900-2019*	Edit
# 17	759	TI=((computer* or television or tv or "video game?" or videogame? or gaming) and (sedentary or "physical* activity*" or sitting or seated or underactiv* or under activ*))<br>*Indexes=SCI-EXPANDED, SSCI, CPCI-S, CPCI-SSH Timespan=1900-2019*	Edit
# 16	3,591	TS= (play* NEAR/5 ("video game*" or "videogame*" or "computer game*"))<br>*Indexes=SCI-EXPANDED, SSCI, CPCI-S, CPCI-SSH Timespan=1900-2019*	Edit
# 15	8,648	TS=((watch* or view*) NEAR/5 (television or tv))<br>*Indexes=SCI-EXPANDED, SSCI, CPCI-S, CPCI-SSH Timespan=1900-2019*	Edit
# 14	62,624	TS=(time NEAR/5 (computer* or television or tv or "video game*" or videogame* or gaming or screen or media))<br>*Indexes=SCI-EXPANDED, SSCI, CPCI-S, CPCI-SSH Timespan=1900-2019*	Edit
# 13	6,328	TS=((decrease or reduc* or discourag* or lessen*) NEAR/3((sit or sitting or stand or standing or "physical* inactiv*")))<br>*Indexes=SCI-EXPANDED, SSCI, CPCI-S, CPCI-SSH Timespan=1900-2019*	Edit
# 12	3,932	TOPIC: (((light or low) near/1 "physical activ*"))<br>*Indexes=SCI-EXPANDED, SSCI, CPCI-S, CPCI-SSH Timespan=1900-2019*	Edit
# 11	978	TS=("sit* less")<br>*Indexes=SCI-EXPANDED, SSCI, CPCI-S, CPCI-SSH Timespan=1900-2019*	Edit
# 10	21,895	TS=((sitting or sit or seated or stationary or standing) NEAR/3 (task* or time or bout* or work* or break*))<br>*Indexes=SCI-EXPANDED, SSCI, CPCI-S, CPCI-SSH Timespan=1900-2019*	Edit
# 9	380	TOPIC: ("chair rise")<br>*Indexes=SCI-EXPANDED, SSCI, CPCI-S, CPCI-SSH Timespan=1900-2019*	Edit
# 8	3,681	TOPIC: ((nonexercis* or "non exercis*" or "no exercis*"))<br>*Indexes=SCI-EXPANDED, SSCI, CPCI-S, CPCI-SSH Timespan=1900-2019*	Edit
# 7	1,800	TOPIC: (((sitting or lying) near/2 posture*))<br>*Indexes=SCI-EXPANDED, SSCI, CPCI-S, CPCI-SSH Timespan=1900-2019*	Edit
# 6	15,258	TS=("physical activity level*" or "physical* inactiv*")<br>*Indexes=SCI-EXPANDED, SSCI, CPCI-S, CPCI-SSH Timespan=1900-2019*	Edit
# 5	3,838	TS=(("leisure time" NEAR/5 ("physical* activ*" or passive or inactiv*)))<br>*Indexes=SCI-EXPANDED, SSCI, CPCI-S, CPCI-SSH Timespan=1900-2019*	Edit
# 4	7,443	TS=((sedentary) near/3 (adult* or men or women or males or females or individuals or people or population*))<br>*Indexes=SCI-EXPANDED, SSCI, CPCI-S, CPCI-SSH Timespan=1900-2019*	Edit
# 3	2,596	TS=((inactive* or "non exercise" or "nonexercise" or "no exercise") near/3 (adult* or men or women or males or females or individuals or people))<br>*Indexes=SCI-EXPANDED, SSCI, CPCI-S, CPCI-SSH Timespan=1900-2019*	Edit
# 2	13,139	TS=(((sedentary or sitting or seated) NEAR/5 (behavio* or lifestyle or life-style)))<br>*Indexes=SCI-EXPANDED, SSCI, CPCI-S, CPCI-SSH Timespan=1900-2019*	Edit
# 1	16,294	TI=((sedentary or sitting or sedentariness or sedentarism))<br>*Indexes=SCI-EXPANDED, SSCI, CPCI-S, CPCI-SSH Timespan=1900-2019*	Edit

ProQuest Dissertations & Theses A&I

		Databases:<br><br>ProQuest Dissertations & Theses A&I 21.3.19		
Select item 1	**S1**	ti((computer* OR television OR tv OR "video game" OR "videogame*" OR gaming) AND (sedentary OR physical* activity* OR sitting OR seated OR underactiv* OR under activ*)) OR ti(sedentary OR sitting OR elementariness OR sedentary OR (sedentary OR sitting OR seated) N5 (behavio* OR lifestyle OR life-style)) OR ti((sitting OR sit OR seated OR stationary OR standing) N3 (task* OR time OR bout* OR work* OR break*)) OR ti("physical* inactiv*" OR "chair rise*" OR "low energy expenditure" OR "sit less") OR ti((watch* OR view*) N5 (television OR tv)) OR ti(play* N5 ("video game*" OR videogame* OR "computer game*")) OR ti(time N5 (computer* OR television OR tv OR "video game*" OR videogame* OR gaming OR screen OR media)) OR ti((computer* OR television OR tv OR "video game" OR "videogame*" OR gaming) AND (sedentary OR physical* activity* OR sitting OR seated OR underactiv* OR "under activ*")) AND ti("process* evaluation*" OR "program* evaluation*") AND ti(Random* OR RCT OR clinical N1 trial*)	ProQuest Dissertations & Theses A&I	**699**
