# Supplementary material for: A protocol for a systematic review of process evaluations of interventions investigating sedentary behaviour in adults
Source: BMJ Open. 2019 Sep 18;9(9):e031291. doi: 10.1136/bmjopen-2019-031291 (PMC6756361; doi:10.1136/bmjopen-2019-031291)
Supplement: Author's manuscript [file bmjopen-2019-031291.draft_revisions.pdf]

BMJ Open is committed to open peer review. As part of this commitment we make the peer review history of every article we publish publicly available.

When an article is published we post the peer reviewers' comments and the authors' responses online. We also post the versions of the paper that were used during peer review. These are the versions that the peer review comments apply to.

The versions of the paper that follow are the versions that were submitted during the peer review process. They are not the versions of record or the final published versions. They should not be cited or distributed as the published version of this manuscript.

BMJ Open is an open access journal and the full, final, typeset and author-corrected version of record of the manuscript is available on our site with no access controls, subscription charges or pay-per-view fees (http://bmjopen.bmj.com).

If you have any questions on BMJ Open's open peer review process please email info.bmjopen@bmj.com

BMJ Open

A protocol for a systematic review of process evaluations of interventions investigating sedentary behaviour in adults

Journal:	*BMJ Open*
Manuscript ID	bmjopen-2019-031291.R1
Article Type:	Protocol
Date Submitted by the Author:	24-Jul-2019
Complete List of Authors:	Corepal, Rekesh; Bradford Royal Infirmary, Academic Unit of Elderly Care and Rehabilitation<br>Hall, Jessica; Bradford Royal Infirmary, Academic Unit of Elderly Care and Rehabilitation<br>English, Coralie; University of Newcastle, School of Health Sciences<br>Farrin, Amanda; University of Leeds, Clinical Trials Research Unit<br>Fitzsimons, Claire F.; University of Edinburgh, ISPEHS<br>Forster, Anne; University of Leeds, Academic Unit of Elderly Care and Rehabilitation<br>Lawton, Rebecca; University of Leeds, Institute of Psychological Sciences; Bradford Institute for Health Research, Quality and Safety Research<br>Mead, Gillian; University of Edinburgh, Geriatric Medicine<br>Clarke, David; University of Leeds, Academic Unit of Elderly Care and Rehabilitation
**Primary Subject Heading**:	Public health
Secondary Subject Heading:	Research methods
Keywords:	Systematic review, Process evaluation, Sedentary behaviour, Protocol

SCHOLARONE™ Manuscripts

Title page

A protocol for a systematic review of process evaluations of interventions investigating sedentary behaviour in adults

Authors:

[1]Rekesh Corepal Rekesh.Corepal@bthft.nhs.uk(corresponding author); [1]Jessica Hall Jessica.Hall@bthft.nhs.uk [2]Coralie English Coralie.English@newcastle.edu.au; [3]Amanda Farrin A.J.Farrin@leeds.ac.uk; [4]Claire Fitzsimons Claire.Fitzsimons@ed.ac.uk; [1]Anne Forster A.Forster@Leeds.ac.uk; [5]Rebecca Lawton R.J.Lawton@leeds.ac.uk; [6]Gillian Mead Gillian.E.Mead@ed.ac.uk; [1]David Clarke D.J.Clarke@leeds.ac.uk.

[1]Academic Unit of Elderly Care and Rehabilitation, Bradford Institute for Health Research, Temple Bank House, Bradford Royal Infirmary, Bradford, BD9 6RJ

[2]Physiotherapy Program, School of Health Sciences, Faculty of Health and Medicine, HC-36 Hunter Building, The University of Newcastle, Australia, NSW 2308

[3]Leeds Institute of Clinical Trials Research, Worsley Building, Clarendon Way, University of Leeds, Leeds, LS2 9NL

[4]Institute for Sport, Physical Education and Health, The University of Edinburgh, St Leonard's land, Holyrood Road, Edinburgh, EH8 8AQ

[5]Yorkshire Quality and Safety, Bradford Institute for Health Research, Temple Bank House, Bradford Royal Infirmary, Bradford, BD9 6RJ

[6]Geriatric Medicine, Division of Health Sciences, Centre for Clinical Brain Sciences, Room F1424, Royal Infirmary, Edinburgh, EH16 4SB

Word count: 2086

For peer review only - http://bmjopen.bmj.com/site/about/guidelines.xhtml

Abstract

Introduction

Sedentary behaviour is defined as any waking behaviour characterised by low energy expenditure ≤1.5 Metabolic Equivalents (METs) while in a sitting, lying or reclining posture. The expanding evidence base suggests that sedentary behaviour may have a detrimental effect on health, wellbeing, and is associated with an increased risk of all-cause mortality.

We aim to review process evaluations of randomised controlled trials (RCTs) which included a measure of sedentary behaviour in adults in order to develop an understanding of intervention content, mechanisms of impact, implementation and delivery approaches and contexts, in which interventions were reported to be effective or effective. A secondary aim is to summarise participants, family, and staff experiences of such interventions.

Methods and analysis:

Ten electronic databases and reference lists from previous similar reviews will be searched. Eligible studies will be process evaluations of RCTs that measure sedentary behaviour as a primary or secondary outcome in adults. As this review will contribute to a programme to develop a community-based intervention to reduce sedentary behaviour in stroke survivors, interventions delivered in schools, colleges, universities or workplaces will be excluded. Two reviewers will perform study selection, data extraction, and quality assessment. Disagreements between reviewers will be resolved by a third reviewer. Process evaluation data to be extracted include: the aims and methods used in the process evaluation; implementation data; mechanisms of impact; contextual factors; participant, family, and staff experiences of the interventions.

For peer review only - http://bmjopen.bmj.com/site/about/guidelines.xhtml

A narrative approach will be used to synthesise and report qualitative and quantitative data. Reporting of the review will be informed by Preferred Reporting Items for Systematic Review and Meta-analysis (PRISMA) guidance.

Ethics and dissemination:

Ethical approval is not required as it is a protocol for a systematic review. Findings will be disseminated through peer-reviewed publications and conference presentations.

Protocol registration number: CRD42018087403

Keywords: Sedentary, Adults, Process evaluation, Systematic review, Protocol

Article Summary

Strengths and limitations of this study

- This systematic review protocol follows the Preferred Reporting Items for Systematic review and Meta-Analysis Protocols (PRISMA-P) guidelines
- This systematic review addresses a gap in the current evidence-base by providing a comprehensive assessment of the implementation, mechanisms of impact, and contextual factors which may influence the effectiveness of RCTs investigating sedentary behaviour in adults
- This review is limited to evidence from randomised trials
- Non-English electronic databases will not be searched. This limitation may cause language bias.
- There is the potential for a low and inconsistent quality in the reporting of process evaluations

For peer review only

For peer review only - http://bmjopen.bmj.com/site/about/guidelines.xhtml

BACKGROUND

Outcome evaluations, such as randomised controlled trials (RCTs), are important to understanding intervention effectiveness, however, in isolation, they may fail to account for how interventions function, why they are successful or not, and for whom.[1] Process evaluations can help to provide this necessary insight.[2] Undertaken alongside outcome evaluations, they include quantitative, qualitative, or mixed-methods approaches *"which aim to understand the functioning of an intervention, by examining implementation, mechanisms of impact and contextual factors".*[3, pg 8]

Process evaluations may also explore the theoretical and logic models informing or underpinning interventions. A theoretical model may be used by researchers in the development of complex interventions to identify key concepts of interest which may be influential in bringing about a desired outcome or change. Logic models are one method of making theoretical assumptions clear, as they graphically illustrate the link between expected outcomes and intervention activities/processes designed to bring about these outcomes.[3 4]

Rationale

Sedentary behaviour is defined as any waking behaviour characterised by low energy expenditure ≤1.5 Metabolic Equivalents (METs) while in a sitting, lying or reclining posture.[5] It has emerged as an important public health issue in the last two decades and has become the focus of considerable clinical, policy and practice research as evidence supporting the detrimental effects of sedentary behaviour on health and wellbeing has increased.[6-8] The negative impact of sedentary behaviour has been highlighted for a number of parameters related to health,[8 9] including reduced physical function,[10 11] increased symptoms of depression,[12] anxiety[13] and cardiovascular risk.[14 15]

The effectiveness of interventions to reduce sedentary behaviour has been synthesised in systematic reviews and meta-analyses.[16-18] However, such work often fails to provide a detailed understanding of the functioning of the interventions.[19] This systematic review of process evaluations aims to fill this gap in the literature.

For peer review only

For peer review only - http://bmjopen.bmj.com/site/about/guidelines.xhtml

This systematic review will contribute to a National Institute for Health Research programme grant for the development and evaluation of strategies to reduce sedentary behaviour in patients after stroke. Currently, there are limited studies looking at reducing sedentary behaviour in stroke survivors, therefore we expanded the search strategy to include all adults to inform a community-based intervention. Although stroke occurs in children and working age adults, the majority of strokes occur in adults aged 65 and over.[20] interventions that take place in schools, colleges, universities and workplaces will be excluded from the review as they are less applicable to our population of interest.

Aims and objectives

Review aim:

To identify and review previously conducted process evaluations of interventions which include a sedentary behaviour outcome measure in adults, in order to develop an understanding of intervention content, mechanisms of impact, implementation and delivery approaches and contexts, in which interventions were reported to be effective or ineffective. A secondary aim is to explore participant, family, and intervention staff experiences in such interventions.

Objectives:

i. To examine the trial data (e.g. design of interventions, sample sizes, duration and content of interventions, and primary and secondary outcome data (from the process evaluation paper or associated papers)..
ii. Establish whether logic models or theoretical models were used to explain how interventions were intended to work.
iii. Establish whether interventions were delivered as intended.
iv. Explore intended or unintended mechanisms of action reported to influence the effectiveness of interventions.
v. Understand barriers and facilitators to delivery of, and participation in, interventions and any recommendations made to address such barriers and facilitators.

For peer review only - http://bmjopen.bmj.com/site/about/guidelines.xhtml

vi. To examine qualitative data concerning the understanding and experiences of interventions from the perspectives of participants, family/carers, and intervention staff.

Qualitative data related to exploring perceptions, views, and lived experiences of sedentary behaviour, but not related to receipt or delivery of an intervention will be transferred to a concurrent qualitative systematic review (Prospero registration number: CRD42017083436).

METHODS AND ANALYSIS

This protocol has been developed following the Preferred Reporting Items for Systematic Review and Meta-analysis Protocols (PRISMA-P) guidelines,[21] as shown in the PRISMA-P checklist (see Additional file 1). The systematic review is prospectively registered with PROSPERO (Prospective Register of Systematic Reviews). Reporting of the systematic review will be informed by Preferred Reporting Items for Systematic Review and Meta-analysis (PRISMA) guidance. [22] Important amendments made to the protocol will be documented and published alongside the results of the systematic review.

Methodological considerations associated with this review

Inclusion and exclusion criteria

Types of studies**:** Studies that are explicitly identified as a process evaluation, or studies that aim to understand the functioning of an intervention by examining implementation, mechanisms of impact, and contextual factors.[3, pg 8] (E.g. implementation processes, patient and staff barriers and facilitators, participants' experiences of delivery or receipt of the intervention).

We will include process evaluations of RCTs that measure sedentary behaviour as an outcome in adults. Process evaluations of feasibility RCTs will be included provided there is random allocation. In process evaluations of cross-over trials, we will only include data from the first phase of the trial. Cohort and uncontrolled before-and-after studies will be excluded.

For peer review only - http://bmjopen.bmj.com/site/about/guidelines.xhtml

Types of Participants: All studies involving adults regardless of whether they were conducted in a clinical or nonclinical population. We will include studies with participants aged 16 or over. We will exclude studies with participants aged less than 16 years of age.

Interventions: Any study which measures sedentary behaviour as an outcome even if reducing sedentary behaviour is not the primary outcome (e.g. moderate-to-vigorous physical activity (MVPA) is the primary outcome).

Interventions that are delivered primarily in schools, colleges, universities or the workplace will be excluded. Studies that do not report any measures of sedentary behaviour as an outcome measure will be excluded. Studies where the main aim is to investigate the acute (immediate) effects of breaking up sitting time as part of a supervised (usually laboratory based) intervention will also be excluded.

Comparators: In the source trial, the intervention group may be compared to: no active treatment, usual care, attention controls waitlist controls, or alternative treatments. Where process evaluations include data from control groups, these data will also be extracted.

Information sources

Electronic searches

In collaboration with information specialist colleagues, informed by guidance from Booth [23] comprehensive search strategies were used using controlled vocabulary and free text terms.

We searched the following electronic databases: CINAHL (EBSCOHost); SPORTDiscus (EBSCOHost); Cochrane Database of Systematic Reviews (Wiley); Cochrane Central Register of Controlled Trials (Wiley): AMED (OVID); EMBASE (OVID); PsycINFO (OVID); Ovid MEDLINE(R); OVID MEDLINE(R) and Epub Ahead of Print, In-Process & Other Non-Indexed Citations; Web of Science: Sciences Citation Index Expanded (Clarivate); Web of Science: Social Sciences Citation Index Expanded (Clarivate); Web of Science: Conference Proceedings Citation

For peer review only

For peer review only - http://bmjopen.bmj.com/site/about/guidelines.xhtml

Index- Science (Clarivate); Web of Science: Conference Proceedings Citation Index- Social Sciences and Humanities (Clarivate); ProQuest Dissertations & Theses A&I.

Searching other relevant sources

In addition to the electronic database searches, we will identify process evaluations through existing systematic reviews of studies of sedentary behaviour interventions. This will include a number of steps:

1. Examining the studies reported within the existing systematic reviews to determine whether they meet the inclusion criteria (e.g. randomised trials, adult population, include an outcome measure of sedentary behaviour).
2. We will read the publication of any studies that meet the criteria in step one to identify any process evaluation work. If a process evaluation is referred to, but no data is reported in these publications, we will:
    i. Match the RCTs to any process evaluations identified through the electronic searches (above).
    ii. If they cannot be matched, we will identify linked published process evaluations by performing citation searching (Google Scholar, PubMed, and Web of Science) and also contact authors of the trial publications to request information on any published or unpublished process evaluations.

The above process will also be reversed to match included process evaluation papers with RCTs. This will allow us to extract findings on the intervention outcomes.

A final search syntax for each electronic database is included in Additional file 2.

Study records

For peer review only - http://bmjopen.bmj.com/site/about/guidelines.xhtml

Data management

We will download references identified in searches (electronic database and additional searches) into Endnote X7 reference management software. Once duplicates are removed, the remaining references will be exported into Covidence (www.covidence.org); an online systematic review tool recommended by the Cochrane Collaboration.

Selection process

The screening process will be undertaken using Covidence. Two review authors will independently assess the titles and abstracts of records and exclude papers that do not meet eligibility criteria. We will obtain the full text of the remaining papers, and two authors will assess the papers against the inclusion criteria for the review to determine their eligibility for inclusion. Non-English language papers will be translated into English. The review authors will resolve disagreements through a consensus-based decision, or if necessary, discussion with a third reviewer.

Data extraction process

Two review authors will independently extract and record data from included studies using a standardised data extraction form. The data extraction form will be guided by the Medical Research Council (MRC) guidance for process evaluations,[1] and previous research which has identified key components for conducting and reporting process evaluations.[24 25] Reviewers will pilot the data extraction form with a sample of included papers and amendments will be made as necessary. After piloting, data extraction will be completed using Covidence. Study authors will be contacted if additional information is required. Following data extraction, two reviewers will aim to resolve any discrepancies by a consensus-based decision, or if necessary, discussion with a third reviewer.

We will extract data about the RCT and the process evaluation. Data to be extracted includes:

1. The trial design and trial information:

For peer review only - http://bmjopen.bmj.com/site/about/guidelines.xhtml

a) The number of participants randomised to each group, and demographic information.

b) The duration and content of what is provided to the intervention group and the comparator group.

c) Primary and secondary outcome results including adverse events measured at post-intervention and follow up.

2. The aims and objectives of the process evaluation and whether the process evaluation was pre-specified or post-hoc.

3. The methods used to conduct the process evaluation.

4. The number of sites sampled for the process evaluation, and sample characteristics (e.g. recruitment and maintenance of participants or participating sites, reach of the intervention into the target population, age, and gender).

5. Implementation data (e.g. what is intended to be delivered? How is delivery achieved? What is delivered? How is adherence measured?

6. Mechanisms of impact (drawing on the logic model or intervention theory used, identified mediators of change, and responses to and interactions with the intervention).

7. Contextual factors that influence implementation, intervention mechanisms, and outcomes.

8. Participants, family/carers, and intervention staff views and experiences of the interventions, including barriers and facilitators. Experiences of control group participants relating to their involvement in the trial.

9. Any conflicts of interest declared by the authors.

Outcomes and prioritisation

To meet our research aims and objectives, the outcomes of interest for this study include the following:

For peer review only - http://bmjopen.bmj.com/site/about/guidelines.xhtml

For peer review only

1. The outcome results from the intervention.
2. Findings from the process data relating to implementation (intended delivery and fidelity to the intervention plan).
3. Adherence to the intervention and how this is measured.
4. Intended and unintended mechanisms of impact was measured.
5. Barriers and facilitators to delivery or participation in the intervention.
6. Adaptations made to improve delivery of the intervention.
7. Participants experiences of the intervention (delivery and receipt).

Findings will clarify key factors that affect intervention delivery and participation. This will provide contextual information useful for explaining why interventions were effective or ineffective, and how interventions could be refined.

Quality assessment

Currently, there is no quality assessment tool designed for judging the quality of process evaluations. Process evaluations can incorporate a combination of both qualitative and quantitative data. Therefore, methodological quality will be evaluated using the Mixed Methods Appraisal Tool (MMAT), which is designed to concurrently assess qualitative, quantitative, and mixed methods studies.[26] Assessment of reporting quality will be guided by Grant et al's framework for reporting process evaluations of cluster randomised controlled trials.[25] Two reviewers will independently assess each study and discrepancies will be resolved by a third reviewer. We will not exclude studies based on findings from the quality assessment.

Data synthesis

Narrative approach to synthesising data

In this review, we will undertake a narrative approach to synthesising data. The synthesis will provide detailed written commentary on the data extracted in accordance with the factors outlined in the 'data collection process' section. This will

For peer review only - http://bmjopen.bmj.com/site/about/guidelines.xhtml

advance our understandings of the intervention context, its delivery, and the mechanisms reported to be effective or ineffective.

Patient and public involvement

As this research will be based on previously published data, there will be no patient and public involvement in the design, interpretation, or dissemination of the findings.

List of abbreviations

AMED: Allied and Complementary Medicine Database

CINAHL: Cumulative Index to Nursing and Allied Health Literature

METS: Metabolic Equivalents

MMAT: Mixed Methods Appraisal Tool

MRC: Medical Research Council

MVPA: Moderate-to Vigorous Physical Activity

NIHR: National Institute for Health Research

PRISMA: Preferred Reporting Items for Systematic Reviews and Meta-Analyses

PRISMA-P: Preferred Reporting Items for Systematic Review and Meta-analysis Protocols

PROSPERO: Prospective Register of Systematic Reviews

RCT: Randomised Controlled Trial

Author Contributions

This systematic review was conceived and designed by members of the Programme Management Group (PMG) of the RECREATE Programme (AF,,CE,AFa,CF,,RL,GM,,DC). This protocol was initially drafted by DC, RC, JH, and AF. Subsequent drafts were commented on by all members of the PMG and revisions made by the authors. All authors have approved submission.

Competing interests

For peer review only - http://bmjopen.bmj.com/site/about/guidelines.xhtml

The authors declare that they have no competing interests.

Acknowledgements

We acknowledge the help and support of our Information Scientist, Deirdre Andre, University of Leeds.

We are grateful for the funding provided by the National Institute for Health Research (NIHR).

Availability of data

Not applicable.

Funding statement

This report is independent research funded by the National Institute for Health Research (Programme Grants for Applied Research, Development and evaluation of strategies to reduce sedentary behaviour in patients after stroke and improve outcomes, RP-PG-0615-20019). The views expressed in this publication are those of the author(s) and not necessarily those of the NIHR or the Department of Health and Social Care.

For peer review only - http://bmjopen.bmj.com/site/about/guidelines.xhtml

References

1. Moore GF, Audrey S, Barker M, et al. Process evaluation of complex interventions: Medical Research Council guidance. *BMJ* 2015;350:h1258. doi: 10.1136/bmj.h1258

2. Craig P, Dieppe P, Macintyre S, et al. Developing and evaluating complex interventions: the new Medical Research Council guidance. *BMJ* 2008;337:a1655. doi: 10.1136/bmj.a1655

3. Moore GF, Audrey S, Barker M, et al. Process evlauations of complex interventions: UK Medical Research Council (MRC) guidance. London: Population Health Sciences Research Network, 2014.

4. Kellogg Foundation W. Using logic models to bring together planning, evaluation, and action: Logic model development guide. *Battle Creek, Mich: WK Kellogg Foundation* 2004

5. Tremblay MS, Aubert S, Barnes JD, et al. Sedentary Behavior Research Network (SBRN) - Terminology Consensus Project process and outcome. *Int J Behav Nutr Phys Act* 2017;14(1):75. doi: 10.1186/s12966-017-0525-8

6. Healy GN, Dunstan DW, Salmon J, et al. Breaks in sedentary time: beneficial associations with metabolic risk. *Diabetes Care* 2008;31(4):661-6. doi: 10.2337/dc07-2046

7. Owen N, Healy GN, Matthews CE, et al. Too much sitting: the population health science of sedentary behavior. *Exerc Sport Sci Rev* 2010;38(3):105-13. doi: 10.1097/JES.0b013e3181e373a2

8. de Rezende LF, Rey-Lopez JP, Matsudo VK, et al. Sedentary behavior and health outcomes among older adults: a systematic review. *BMC Public Health* 2014;14:333. doi: 10.1186/1471-2458-14-333

9. Wullems JA, Verschueren SM, Degens H, et al. A review of the assessment and prevalence of sedentarism in older adults, its physiology/health impact and non-exercise mobility counter-measures. *Biogerontology* 2016;17(3):547-65. doi: 10.1007/s10522-016-9640-1

10. Gennuso KP, Gangnon RE, Matthews CE, et al. Sedentary behavior, physical activity, and markers of health in older adults. *Med Sci Sports Exerc* 2013;45(8):1493-500. doi: 10.1249/MSS.0b013e318288a1e5

11. Santos DA, Silva AM, Baptista F, et al. Sedentary behavior and physical activity are independently related to functional fitness in older adults. *Exp Gerontol* 2012;47(12):908-12. doi: 10.1016/j.exger.2012.07.011

12. Teychenne M, Ball K, Salmon J. Sedentary behavior and depression among adults: a review. *Int J Behav Med* 2010;17(4):246-54. doi: 10.1007/s12529-010-9075-z

For peer review only - http://bmjopen.bmj.com/site/about/guidelines.xhtml

For peer review only

13. Teychenne M, Costigan SA, Parker K. The association between sedentary behaviour and risk of anxiety: a systematic review. *BMC Public Health* 2015;15:513. doi: 10.1186/s12889-015-1843-x

14. Edwardson CL, Gorely T, Davies MJ, et al. Association of sedentary behaviour with metabolic syndrome: a meta-analysis. *PLoS One* 2012;7(4):e34916. doi: 10.1371/journal.pone.0034916

15. Same RV, Feldman DI, Shah N, et al. Relationship Between Sedentary Behavior and Cardiovascular Risk. *Curr Cardiol Rep* 2016;18(1):6. doi: 10.1007/s11886-015-0678-5

16. Shrestha N, Grgic J, Wiesner G, et al. Effectiveness of interventions for reducing non-occupational sedentary behaviour in adults and older adults: a systematic review and meta-analysis. *Br J Sports Med* 2018 doi: 10.1136/bjsports-2017-098270

17. Martin A, Fitzsimons C, Jepson R, et al. Interventions with potential to reduce sedentary time in adults: systematic review and meta-analysis. *Br J Sports Med* 2015;49(16):1056-63. doi: 10.1136/bjsports-2014-094524

18. Thraen-Borowski KM, Ellingson LD, Meyer JD, et al. Nonworksite Interventions to Reduce Sedentary Behavior among Adults: A Systematic Review. *Transl J Am Coll Sports Med* 2017;2(12):68-78. doi: 10.1249/TJX.0000000000000036

19. Linnan L, Steckler A. Process evaluation for public health interventions and research: Jossey-Bass 2002.

20. Roy-O'Reilly M, McCullough LD. Age and Sex Are Critical Factors in Ischemic Stroke Pathology. *Endocrinology* 2018;159(8):3120-31. doi: 10.1210/en.2018-00465

21. Moher D, Shamseer L, Clarke M, et al. Preferred reporting items for systematic review and meta-analysis protocols (PRISMA-P) 2015 statement. *Syst Rev* 2015;4:1. doi: 10.1186/2046-4053-4-1

22. Moher D, Liberati A, Tetzlaff J, et al. Preferred reporting items for systematic reviews and meta-analyses: the PRISMA statement. *BMJ* 2009;339:b2535. doi: 10.1136/bmj.b2535

23. Booth A. Searching for studies. In: Noyes J, Booth A, Hannes K, et al., eds. Supplementary Guidance for Inclusion of Qualitative Research in Cochrane Systematic Reviews of Interventions: Cochrane Collaboration Qualitative Methods Group, 2011.

24. Baranowski T, Stables G. Process evaluations of the 5-a-day projects. *Health Educ Behav* 2000;27(2):157-66. doi: 10.1177/109019810002700202

For peer review only - http://bmjopen.bmj.com/site/about/guidelines.xhtml

25. Grant A, Treweek S, Dreischulte T, et al. Process evaluations for cluster-randomised trials of complex interventions: a proposed framework for design and reporting. *Trials* 2013;14:15. doi: 10.1186/1745-6215-14-15

26. Pace R, Pluye P, Bartlett G, et al. Testing the reliability and efficiency of the pilot Mixed Methods Appraisal Tool (MMAT) for systematic mixed studies review. *Int J Nurs Stud* 2012;49(1):47-53. doi: 10.1016/j.ijnurstu.2011.07.002

For peer review only

Additional file 1: PRISMA –P checklist

PRISMA-P (Preferred Reporting Items for Systematic review and Meta-Analysis Protocols) 2015 checklist: recommended items to address in a systematic review protocol*

Section and topic	Item #	Checklist item	Page #
**ADMINISTRATIVE INFORMATION**			
Title:			
Identification	1a	Identify the report as a protocol of a systematic review	1,6
Update	1b	If the protocol is for an update of a previous systematic review, identify as such	N/A*
Registration	2	If registered, provide the name of the registry (such as PROSPERO) and registration number	3
Authors:			
Contact	3a	Provide name, institutional affiliation, e-mail address of all protocol authors; provide physical mailing address of corresponding author	1
Contributions	3b	Describe contributions of protocol authors and identify the guarantor of the review	14
Amendments	4	If the protocol represents an amendment of a previously completed or published protocol, identify as such and list changes; otherwise, state plan for documenting important protocol amendments	6
Support:			
Sources	5a	Indicate sources of financial or other support for the review	14
Sponsor	5b	Provide name for the review funder and/or sponsor	14
Role of sponsor or funder	5c	Describe roles of funder(s), sponsor(s), and/or institution(s), if any, in developing the protocol	14

For peer review only

**INTRODUCTION**			
Rationale	6	Describe the rationale for the review in the context of what is already known	4
Objectives	7	Provide an explicit statement of the question(s) the review will address with reference to participants, interventions, comparators, and outcomes (PICO)	5
**METHODS**			
Eligibility criteria	8	Specify the study characteristics (such as PICO, study design, setting, time frame) and report characteristics (such as years considered, language, publication status) to be used as criteria for eligibility for the review	6-7
Information sources	9	Describe all intended information sources (such as electronic databases, contact with study authors, trial registers or other grey literature sources) with planned dates of coverage	7-8
Search strategy	10	Present draft of search strategy to be used for at least one electronic database, including planned limits, such that it could be repeated	8 Additional file 2
Study records:			
Data management	11a	Describe the mechanism(s) that will be used to manage records and data throughout the review	9
Selection process	11b	State the process that will be used for selecting studies (such as two independent reviewers) through each phase of the review (that is, screening, eligibility and inclusion in meta-analysis)	9
Data collection process	11c	Describe planned method of extracting data from reports (such as piloting forms, done independently, in duplicate), any processes for obtaining and confirming data from investigators	9
Data items	12	List and define all variables for which data will be sought (such as PICO items, funding sources), any pre-planned data assumptions and simplifications	10-11
Outcomes and prioritization	13	List and define all outcomes for which data will be sought, including prioritization of main and additional outcomes, with rationale	10-11

For peer review only

Risk of bias in individual studies	14	Describe anticipated methods for assessing risk of bias of individual studies, including whether this will be done at the outcome or study level, or both; state how this information will be used in data synthesis	11
Data synthesis	15a	Describe criteria under which study data will be quantitatively synthesised	9
	15b	If data are appropriate for quantitative synthesis, describe planned summary measures, methods of handling data and methods of combining data from studies, including any planned exploration of consistency (such as I^2, Kendall's τ)	N/A
	15c	Describe any proposed additional analyses (such as sensitivity or subgroup analyses, meta-regression)	N/A
	15d	If quantitative synthesis is not appropriate, describe the type of summary planned	12
Meta-bias(es)	16	Specify any planned assessment of meta-bias(es) (such as publication bias across studies, selective reporting within studies)	N/A
Confidence in cumulative evidence	17	Describe how the strength of the body of evidence will be assessed (such as GRADE)	N/A

[*] **N/A = Not applicable**

From: Shamseer L, Moher D, Clarke M, Ghersi D, Liberati A, Petticrew M, Shekelle P, Stewart L, PRISMA-P Group. Preferred reporting items for systematic review and meta-analysis protocols (PRISMA-P) 2015: elaboration and explanation. BMJ. 2015 Jan 2;349(jan02 1):g7647.

For peer review only

Additional file 2: Search strategies

CINHAL (EBSCOHost)

#	Query	Limiters/Expanders	Last Run Via	Results	Action
S40	S37 AND S38 AND S39	Search modes - Boolean/Phrase	Interface - EBSCOhost Research Databases Search Screen - Advanced Search Database - CINAHL	289	
S39	S33 OR S34 OR S35 or S36	Search modes - Boolean/Phrase	Interface - EBSCOhost Research Databases Search Screen - Advanced Search Database - CINAHL	56,168	
S38	S21 OR S22 OR S23 OR S24 OR S25 OR S26 OR S27 OR S28 OR S29 OR S30 OR S31	Search modes - Boolean/Phrase	Interface - EBSCOhost Research Databases Search Screen - Advanced Search Database - CINAHL	435,132	
S37	S1 OR S2 OR S3 OR S4 OR S5 OR S6 OR S7 OR S8 OR S9 OR S10 OR S11 OR S12 OR S13 OR S14 OR S15 OR S16 OR S17 OR S18 OR S19 OR S20	Search modes - Boolean/Phrase	Interface - EBSCOhost Research Databases Search Screen - Advanced Search Database - CINAHL	29,934	
S36	(MH "Process Assessment (Health Care)")	Search modes - Boolean/Phrase	Interface - EBSCOhost Research Databases Search Screen - Advanced Search Database - CINAHL	4,433	
S35	TX ((program* evaluat*))	Search modes - Boolean/Phrase	Interface - EBSCOhost Research Databases Search Screen - Advanced Search Database - CINAHL	52,056	

For peer review only

S34	Tx ((process* evaluat*))	Search modes - Boolean/Phrase	Interface - EBSCOhost Research Databases Search Screen - Advanced Search Database - CINAHL	1
S33	(MH "Program Evaluation")	Search modes - Boolean/Phrase	Interface - EBSCOhost Research Databases Search Screen - Advanced Search Database - CINAHL	34,068
S32	S21 or S22 or S23 or S24 or S25 or S26 or S27 or S28 or S29 or S30 or S31	Search modes - Boolean/Phrase	Interface - EBSCOhost Research Databases Search Screen - Advanced Search Database - CINAHL	435,132
S31	MH "Random Assignment"	Search modes - Boolean/Phrase	Interface - EBSCOhost Research Databases Search Screen - Advanced Search Database - CINAHL	53,614
S30	AB randomly	Search modes - Boolean/Phrase	Interface - EBSCOhost Research Databases Search Screen - Advanced Search Database - CINAHL	70,962
S29	AB randomized	Search modes - Boolean/Phrase	Interface - EBSCOhost Research Databases Search Screen - Advanced Search Database - CINAHL	129,654
S28	(MH "Clinical Trials+")	Search modes - Boolean/Phrase	Interface - EBSCOhost Research Databases Search Screen - Advanced Search Database - CINAHL	254,465
S27	PT Clinical trial	Search modes - Boolean/Phrase	Interface - EBSCOhost Research Databases Search Screen - Advanced Search Database - CINAHL	86,264

For peer review only

S26	TX clinic* n1 trial*	Search modes - Boolean/Phrase	Interface - EBSCOhost Research Databases Search Screen - Advanced Search Database - CINAHL	238,716
S25	TX randomi* control* trial*	Search modes - Boolean/Phrase	Interface - EBSCOhost Research Databases Search Screen - Advanced Search Database - CINAHL	164,354
S24	TX random* allocat*	Search modes - Boolean/Phrase	Interface - EBSCOhost Research Databases Search Screen - Advanced Search Database - CINAHL	9,911
S23	TX placebo*	Search modes - Boolean/Phrase	Interface - EBSCOhost Research Databases Search Screen - Advanced Search Database - CINAHL	55,417
S22	(MH "Placebos")	Search modes - Boolean/Phrase	Interface - EBSCOhost Research Databases Search Screen - Advanced Search Database - CINAHL	11,050
S21	TX allocat* random*	Search modes - Boolean/Phrase	Interface - EBSCOhost Research Databases Search Screen - Advanced Search Database - CINAHL	9,911
S20	TI (play* N5 ("video game*" or videogame* or "computer game*")) OR AB (play* N5 ("video game*" or videogame* or "computer game*"))	Search modes - Boolean/Phrase	Interface - EBSCOhost Research Databases Search Screen - Advanced Search Database - CINAHL	706
S19	TX ((watch* or view*) N5 (television	Search modes - Boolean/Phrase	Interface - EBSCOhost Research Databases Search Screen -	2,643

For peer review only

	or tv))		Advanced Search Database - CINAHL	
S18	TX (time N5 (computer* or television or tv or "video game*" or videogame* or gaming or screen or media))	Search modes - Boolean/Phrase	Interface - EBSCOhost Research Databases Search Screen - Advanced Search Database - CINAHL	4,338
S17	TX ((decrease or reduc* or discourag* or lessen*) N3 (sit or sitting or stand or standing or "physical* inactiv*"))	Search modes - Boolean/Phrase	Interface - EBSCOhost Research Databases Search Screen - Advanced Search Database - CINAHL	653
S16	TX ((light or low) N1 "physical activ*")	Search modes - Boolean/Phrase	Interface - EBSCOhost Research Databases Search Screen - Advanced Search Database - CINAHL	1,425
S15	TX "sit* less"	Search modes - Boolean/Phrase	Interface - EBSCOhost Research Databases Search Screen - Advanced Search Database - CINAHL	192
S14	TX "chair rise*"	Search modes - Boolean/Phrase	Interface - EBSCOhost Research Databases Search Screen - Advanced Search Database - CINAHL	234
S13	TI((computer* or television or tv or video game? or videogame? or gaming) and (sedentary or "physical* activity*" or sitting or seated or underactiv* or under activ*))	Search modes - Boolean/Phrase	Interface - EBSCOhost Research Databases Search Screen - Advanced Search Database - CINAHL	274

For peer review only

S12	TX (prolong* N2 (reclin* or sit or sitting or seated))	Search modes - Boolean/Phrase	Interface - EBSCOhost Research Databases Search Screen - Advanced Search Database - CINAHL	432
S11	TX ((sitting or lying) N2 posture*)	Search modes - Boolean/Phrase	Interface - EBSCOhost Research Databases Search Screen - Advanced Search Database - CINAHL	574
S10	TX "physical activity level*"	Search modes - Boolean/Phrase	Interface - EBSCOhost Research Databases Search Screen - Advanced Search Database - CINAHL	4,119
S9	TX (leisure time N5 ("physical* activ*" or passive or inactiv*)))	Search modes - Boolean/Phrase	Interface - EBSCOhost Research Databases Search Screen - Advanced Search Database - CINAHL	2,195
S8	TX "physical* inactiv*"	Search modes - Boolean/Phrase	Interface - EBSCOhost Research Databases Search Screen - Advanced Search Database - CINAHL	3,605
S7	TX "low energy expenditure"	Search modes - Boolean/Phrase	Interface - EBSCOhost Research Databases Search Screen - Advanced Search Database - CINAHL	55
S6	TX((inactiv* or no exercise or nonexercise or non exercise) N3 (adult* or men or women or males or females or individuals or people))	Search modes - Boolean/Phrase	Interface - EBSCOhost Research Databases Search Screen - Advanced Search Database - CINAHL	1,532
S5	TI (((sitting or sit or seated or stationary or standing) N3	Search modes - Boolean/Phrase	Interface - EBSCOhost Research Databases Search Screen -	2,598

For peer review only

	(task* or time or bout* or work* or break*))) OR AB (((sitting or sit or seated or stationary or standing) N3 (task* or time or bout* or work* or break*)))		Advanced Search Database - CINAHL	
S4	TX ((sedentary N3 (adult* or men or women or males or females or individuals or people or population*))	Search modes - Boolean/Phrase	Interface - EBSCOhost Research Databases Search Screen - Advanced Search Database - CINAHL	2,713
S3	TX ((sedentary or sitting or seated) N5 (behavio* or lifestyle or life-style)))	Search modes - Boolean/Phrase	Interface - EBSCOhost Research Databases Search Screen - Advanced Search Database - CINAHL	9,170
S2	TI (sedentary or sitting or sedentariness or sedentarism)	Search modes - Boolean/Phrase	Interface - EBSCOhost Research Databases Search Screen - Advanced Search Database - CINAHL	4,477
S1	(MH "Life Style, Sedentary")	Search modes - Boolean/Phrase	Interface - EBSCOhost Research Databases Search Screen - Advanced Search Database - CINAHL	6,680

For peer review only

SPORTDiscus (EBSCOHost)

	Query	Limiters/Expanders	Last Run Via	Results	Action
S39	S33 AND S37 AND S38	Search modes - Boolean/Phrase	Interface - EBSCOhost Research Databases Search Screen - Advanced Search Database - SPORTDiscus	37	
S38	S1 OR S2 OR S3 OR S4 OR S5 OR S6 OR S7 OR S8 OR S9 OR S10 OR S11 OR S12 OR S13 OR S14 OR S15 OR S16 OR S17 OR S18 OR S19 OR S20	Search modes - Boolean/Phrase	Interface - EBSCOhost Research Databases Search Screen - Advanced Search Database - SPORTDiscus	19,478	
S37	S34 OR S35 OR S36	Search modes - Boolean/Phrase	Interface - EBSCOhost Research Databases Search Screen - Advanced Search Database - SPORTDiscus	5,377	
S36	TI process* evaluat*	Search modes - Boolean/Phrase	Interface - EBSCOhost Research Databases Search Screen - Advanced Search Database - SPORTDiscus	295	
S35	TX program* evaluat*	Search modes - Boolean/Phrase	Interface - EBSCOhost Research Databases Search Screen - Advanced Search Database - SPORTDiscus	5,148	
S34	SU program evaluation	Search modes - Boolean/Phrase	Interface - EBSCOhost Research Databases Search Screen - Advanced Search Database - SPORTDiscus	1,209	
S33	S21 OR S22 OR S23 OR S24 OR S25 OR S26 OR S27 OR S28 OR S29 OR S30 OR	Search modes - Boolean/Phrase	Interface - EBSCOhost Research Databases Search Screen - Advanced Search	52,680	

For peer review only

	S31 OR S32		Database - SPORTDiscus	
S32	AB randomized	Search modes - Boolean/Phrase	Interface - EBSCOhost Research Databases Search Screen - Advanced Search Database - SPORTDiscus	19,923
S31	AB randomly	Search modes - Boolean/Phrase	Interface - EBSCOhost Research Databases Search Screen - Advanced Search Database - SPORTDiscus	15,478
S30	DE "CLINICAL trials"	Search modes - Boolean/Phrase	Interface - EBSCOhost Research Databases Search Screen - Advanced Search Database - SPORTDiscus	8,795
S29	TX clinic* n1 trial*	Search modes - Boolean/Phrase	Interface - EBSCOhost Research Databases Search Screen - Advanced Search Database - SPORTDiscus	15,367
S28	TX randomi* control* trial*	Search modes - Boolean/Phrase	Interface - EBSCOhost Research Databases Search Screen - Advanced Search Database - SPORTDiscus	17,436
S27	TX random* assign*	Search modes - Boolean/Phrase	Interface - EBSCOhost Research Databases Search Screen - Advanced Search Database - SPORTDiscus	8,031
S26	TX random* allocat*	Search modes - Boolean/Phrase	Interface - EBSCOhost Research Databases Search Screen - Advanced Search Database - SPORTDiscus	1,576
S25	TX placebo*	Search modes - Boolean/Phrase	Interface - EBSCOhost Research Databases Search Screen -	10,556

For peer review only

			Advanced Search Database - SPORTDiscus	
S24	DE "PLACEBOS (Medicine)"	Search modes - Boolean/Phrase	Interface - EBSCOhost Research Databases Search Screen - Advanced Search Database - SPORTDiscus	2,412
S23	DE "QUANTITATIVE research"	Search modes - Boolean/Phrase	Interface - EBSCOhost Research Databases Search Screen - Advanced Search Database - SPORTDiscus	1,448
S22	TX allocat* random*	Search modes - Boolean/Phrase	Interface - EBSCOhost Research Databases Search Screen - Advanced Search Database - SPORTDiscus	1,576
S21	((DE "RANDOMIZED controlled trials")))	Search modes - Boolean/Phrase	Interface - EBSCOhost Research Databases Search Screen - Advanced Search Database - SPORTDiscus	11,047
S20	TI (play* N5 ("video game*" or videogame* or "computer game*")) OR AB (play* N5 ("video game*" or videogame* or "computer game*"))	Search modes - Boolean/Phrase	Interface - EBSCOhost Research Databases Search Screen - Advanced Search Database - SPORTDiscus	564
S19	TI ((watch* or view*) N5 (television or tv)) OR AB ((watch* or view*) N5 (television or tv))	Search modes - Boolean/Phrase	Interface - EBSCOhost Research Databases Search Screen - Advanced Search Database - SPORTDiscus	2,377
S18	TI (time N5 (computer* or television or tv or "video game*" or videogame* or gaming or screen or	Search modes - Boolean/Phrase	Interface - EBSCOhost Research Databases Search Screen - Advanced Search Database - SPORTDiscus	1,711

	media)) OR AB (time N5 (computer* or television or tv or "video game*" or videogame* or gaming or screen or media))			
S17	TI ((decrease or reduc* or discourag* or lessen*) N3 (sit or sitting or stand or standing or "physical* inactiv*")) OR AB ((decrease or reduc* or discourag* or lessen*) N3 (sit or sitting or stand or standing or "physical* inactiv*"))	Search modes - Boolean/Phrase	Interface - EBSCOhost Research Databases Search Screen - Advanced Search Database - SPORTDiscus	303
S16	TI ((light or low) N1 "physical activ*") OR AB ((light or low) N1 "physical activ*")	Search modes - Boolean/Phrase	Interface - EBSCOhost Research Databases Search Screen - Advanced Search Database - SPORTDiscus	614
S15	TI "sit* less" OR AB "sit* less"	Search modes - Boolean/Phrase	Interface - EBSCOhost Research Databases Search Screen - Advanced Search Database - SPORTDiscus	41
S14	TI "chair rise*" OR AB "chair rise*"	Search modes - Boolean/Phrase	Interface - EBSCOhost Research Databases Search Screen - Advanced Search Database - SPORTDiscus	75
S13	TX((computer* or television or tv or video game? or videogame? or gaming) and (sedentary or physical* activity* or sitting or seated or underactiv* or under	Search modes - Boolean/Phrase	Interface - EBSCOhost Research Databases Search Screen - Advanced Search Database - SPORTDiscus	2,498

For peer review only

	activ*))			
S12	TI (prolong* N2 (reclin* or sit or sitting or seated)) OR AB (prolong* N2 (reclin* or sit or sitting or seated))	Search modes - Boolean/Phrase	Interface - EBSCOhost Research Databases Search Screen - Advanced Search Database - SPORTDiscus	203
S11	TI ((sitting or lying) N2 posture*) OR AB ((sitting or lying) N2 posture*)	Search modes - Boolean/Phrase	Interface - EBSCOhost Research Databases Search Screen - Advanced Search Database - SPORTDiscus	321
S10	TI "physical activity level*" OR AB "physical activity level*"	Search modes - Boolean/Phrase	Interface - EBSCOhost Research Databases Search Screen - Advanced Search Database - SPORTDiscus	2,795
S9	TI (leisure time N5 ("physical* activ*" or passive or inactiv*))) OR AB (leisure time N5 ("physical* activ*" or passive or inactiv*))	Search modes - Boolean/Phrase	Interface - EBSCOhost Research Databases Search Screen - Advanced Search Database - SPORTDiscus	1,268
S8	TI "physical* inactiv*" OR AB "physical* inactiv*"	Search modes - Boolean/Phrase	Interface - EBSCOhost Research Databases Search Screen - Advanced Search Database - SPORTDiscus	1,626
S7	TI "low energy expenditure" OR AB "low energy expenditure"	Search modes - Boolean/Phrase	Interface - EBSCOhost Research Databases Search Screen - Advanced Search Database - SPORTDiscus	25
S6	TX(inactiv* or no exercise or nonexercise or non exercise) N3 (adult* or men or women or males or females or individuals or	Search modes - Boolean/Phrase	Interface - EBSCOhost Research Databases Search Screen - Advanced Search Database - SPORTDiscus	1,073

	people))			
S5	TI (((sitting or sit or seated or stationary or standing) N3 (task* or time or bout* or work* or break*))) OR AB (((sitting or sit or seated or stationary or standing) N3 (task* or time or bout* or work* or break*)))	Search modes - Boolean/Phrase	Interface - EBSCOhost Research Databases Search Screen - Advanced Search Database - SPORTDiscus	1,733
S4	TI ((sedentary N3 (adult* or men or women or males or females or individuals or people or population*))) OR AB ((sedentary N3 (adult* or men or women or males or females or individuals or people or population*)))	Search modes - Boolean/Phrase	Interface - EBSCOhost Research Databases Search Screen - Advanced Search Database - SPORTDiscus	2,922
S3	TI ((sedentary or sitting or seated) N5 (behavio* or lifestyle or life-style))) OR AB (seated) N5 (behavio* or lifestyle or life-style)) OR AB ((sedentary or sitting or seated) N5 (behavio* or lifestyle or life-style))) OR AB (seated) N5 (behavio* or lifestyle or life-style))	Search modes - Boolean/Phrase	Interface - EBSCOhost Research Databases Search Screen - Advanced Search Database - SPORTDiscus	2,419
S2	TI (sedentary or sitting or sedentariness or sedentarism)	Search modes - Boolean/Phrase	Interface - EBSCOhost Research Databases Search Screen - Advanced Search Database - SPORTDiscus	3,110

For peer review only

S1	SU Sedentary Lifestyle	Search modes - Boolean/Phrase	Interface - EBSCOhost Research Databases<br>Search Screen - Advanced Search<br>Database - SPORTDiscus	

For peer review only

Cochrane Database of Systematic Reviews (Wiley)

#1 MeSH descriptor: [Sedentary Behavior] this term only 906

#2 sedentary or sitting or sedentariness or sedentarism:ti 9230

#3 (sedentary or sitting or seated) near/5 (behavio* or lifestyle or life-style):ti,ab,kw (Word variations have been searched) 2297

#4 sedentary near/3 (adult* or men or women or males or females or individuals or people or population*):ti,ab,kw (Word variations have been searched) 1920

#5 (sitting or sit or seated or stationary or standing) near/3 (task* or time or bout* or work* or break*):ti,ab,kw (Word variations have been searched) 1265

#6 ((inactiv* or no exercise or nonexercise or non exercise) near/3 (adult* or men or women or males or females or individuals or people)):ti,ab,kw 13841

#7 "low energy expenditure":ti,ab,kw (Word variations have been searched) 10

#8 "physical* inactiv*":ti,ab,kw (Word variations have been searched) 0

#9 "leisure time" near/5 ("physical* activ*" or passive or inactiv*):ti,ab,kw (Word variations have been searched) 224

#10 "physical activity level*":ti,ab,kw (Word variations have been searched) 1459

#11 (sitting or lying) near/2 posture*:ti,ab,kw (Word variations have been searched) 138

#12 prolong* near/2 (reclin* or sit or sitting or seated):ti,ab,kw (Word variations have been searched) 183

#13 "chair rise*":ti,ab,kw (Word variations have been searched) 178

#14 "sit* less":ti,ab,kw (Word variations have been searched) 30

#15 (light or low) near/1 "physical activ*":ti,ab,kw (Word variations have been searched) 226

#16 time near/5 (computer* or television or tv or "video game*" or videogame* or gaming or screen or media):ti,ab,kw (Word variations have been searched) 2056

#17 (watch* or view*) near/5 (television or tv):ti,ab,kw (Word variations have been searched) 464

#18 play* near/5 ("video game*" or videogame* or "computer game*"):ti,ab,kw (Word variations have been searched) 291

#19 (decrease or reduc* or discourag* or lessen*) near/3 (sit or sitting or stand or standing or "physical* inactiv*"):ti,ab,kw (Word variations have been searched) 751

#20 ((computer* or television or tv or video game* or videogame* or gaming) and (sedentary or physical* activity* or sitting or seated or underactiv* or under activ*)):ti 124

#21 {or #1-#20} 27029

#22 MeSH descriptor: [Program Evaluation] this term only 5548

#23 "program* evaluation*":ti,ab,kw 6047

For peer review only

#24	"process* evaluation*":ti,ab,kw	1318
#25	MeSH descriptor: [Process Assessment (Health Care)] this term only	193
#26	{or #22-#25}	7251
#27	#21 and #26	483

For peer review only

Cochrane Central Register of Controlled Trials (Wiley)

#1 MeSH descriptor: [Sedentary Behavior] this term only 906

#2 sedentary or sitting or sedentariness or sedentarism:ti 9230

#3 (sedentary or sitting or seated) near/5 (behavio* or lifestyle or life-style):ti,ab,kw (Word variations have been searched) 2297

#4 sedentary near/3 (adult* or men or women or males or females or individuals or people or population*):ti,ab,kw (Word variations have been searched) 1920

#5 (sitting or sit or seated or stationary or standing) near/3 (task* or time or bout* or work* or break*):ti,ab,kw (Word variations have been searched) 1265

#6 ((inactiv* or no exercise or nonexercise or non exercise) near/3 (adult* or men or women or males or females or individuals or people)):ti,ab,kw 13841

#7 "low energy expenditure":ti,ab,kw (Word variations have been searched) 10

#8 ("physical* inactive" or "physical inactivity"):ti,ab,kw (Word variations have been searched) 987

#9 "leisure time" near/5 ("physical* activ*" or passive or inactiv*):ti,ab,kw (Word variations have been searched) 224

#10 "physical activity level*":ti,ab,kw (Word variations have been searched) 1459

#11 (sitting or lying) near/2 posture*:ti,ab,kw (Word variations have been searched) 138

#12 prolong* near/2 (reclin* or sit or sitting or seated):ti,ab,kw (Word variations have been searched) 183

#13 "chair rise*":ti,ab,kw (Word variations have been searched) 178

#14 "sit* less":ti,ab,kw (Word variations have been searched) 30

#15 (light or low) near/1 "physical activ*":ti,ab,kw (Word variations have been searched) 226

#16 time near/5 (computer* or television or tv or "video game*" or videogame* or gaming or screen or media):ti,ab,kw (Word variations have been searched) 2056

#17 (watch* or view*) near/5 (television or tv):ti,ab,kw (Word variations have been searched) 464

#18 play* near/5 ("video game*" or videogame* or "computer game*"):ti,ab,kw (Word variations have been searched) 291

#19 (decrease or reduc* or discourag* or lessen*) near/3 (sit or sitting or stand or standing or "physical* inactiv*"):ti,ab,kw (Word variations have been searched) 751

#20 ((computer* or television or tv or video game* or videogame* or gaming) and (sedentary or physical* activity* or sitting or seated or underactiv* or under activ*)):ti 124

#21 {or #1-#20} 27534

#22 MeSH descriptor: [Program Evaluation] this term only 5548

#23 ("program* evaluation*"):ti,ab,kw6047

For peer review only

#24	"process* evaluation*":ti,ab,kw	1318
#25	MeSH descriptor: [Process Assessment (Health Care)] this term only	193
#26	{or #22-#25}	7251
#27	#21 and #26 in Trials	486

For peer review only

AMED (Allied and Complementary Medicine)

1 Sedentary Lifestyle/ (292)

2 (sedentary or sitting or sedentariness or sedentarism).ti. (702)

3 ((sedentary or sitting or seated) adj5 (behavio* or lifestyle or life-style)).tw. (471)

4 ((inactiv* or no exercise or nonexercise or non exercise) adj3 (adult? or men or women or males or females or individuals or people)).tw. (1509)

5 (sedentary adj3 (adult? or men or women or males or females or individuals or people or population?)).tw. (382)

6 ((sitting or sit or seated or stationary or standing) adj3 (task* or time or bout* or work* or break*)).tw. (563)

7 low energy expenditure.tw. (4)

8 physical* inactiv*.tw. (216)

9 (leisure time adj5 (physical* activ* or passive or inactiv*)).tw. (152)

10 "physical activity level*".tw. (405)

11 ((sitting or lying) adj2 posture*).tw. (210)

12 (prolong* adj2 (reclin* or sit or sitting or seated)).tw. (56)

13 chair rise?.tw. (58)

14 "sit* less".tw. (13)

15 ((light or low) adj "physical activ*").tw. (48)

16 ((decrease or reduc* or discourag* or lessen*) adj3 (sit or sitting or stand or standing or physical* inactiv*)).tw. (95)

17 (time adj5 (computer* or television or tv or video game? or videogame? or gaming or screen or media)).tw. (189)

18 ((watch* or view*) adj5 (television or tv)).tw. (69)

19 (play* adj5 (video game? or videogame? or computer game?)).tw. (47)

20 ((computer* or television or tv or video game? or videogame? or gaming) and (sedentary or physical* activity* or sitting or seated or underactiv* or under activ*)).ti. (99)

21 or/1-20 [sedentary behaviour terms] (4197)

22 process evaluat*.mp. (88)

23 "Outcome and Process Assessment"/ (1147)

24 program evaluat*.mp. (2347)

25 or/22-24 [process evaluation] (3523)

26 21 and 25 [sedentary behaviour and process evaluation] (76)

For peer review only

EMBASE (OVID)

1 Sedentary Lifestyle/ (11663)

2 (sedentary or sitting or sedentariness or sedentarism).ti. (7958)

3 ((sedentary or sitting or seated) adj5 (behavio* or lifestyle or life-style)).tw. (11608)

4 ((inactiv* or no exercise or nonexercise or non exercise) adj3 (adult? or men or women or males or females or individuals or people)).tw. (3191)

5 (sedentary adj3 (adult? or men or women or males or females or individuals or people or population?)).tw. (6088)

6 ((sitting or sit or seated or stationary or standing) adj3 (task* or time or bout* or work* or break*)).tw. (6389)

7 low energy expenditure.tw. (196)

8 physical* inactiv*.tw. (10206)

9 (leisure time adj5 (physical* activ* or passive or inactiv*)).tw. (4182)

10 "physical activity level*".tw. (10320)

11 ((sitting or lying) adj2 posture*).tw. (1181)

12 (prolong* adj2 (reclin* or sit or sitting or seated)).tw. (820)

13 chair rise?.tw. (561)

14 "sit* less".tw. (1080)

15 ((light or low) adj "physical activ*").tw. (3123)

16 ((decrease or reduc* or discourag* or lessen*) adj3 (sit or sitting or stand or standing or physical* inactiv*)).tw. (1570)

17 (time adj5 (computer* or television or tv or video game? or videogame? or gaming or screen or media)).tw. (12058)

18 ((watch* or view*) adj5 (television or tv)).tw. (5775)

19 (play* adj5 (video game? or videogame? or computer game?)).tw. (1941)

20 ((computer* or television or tv or video game? or videogame? or gaming) and (sedentary or physical* activity* or sitting or seated or underactiv* or under activ*)).ti. (432)

21 or/1-20 [sedentary behaviour terms] (72983)

22 Randomized controlled trial/ (489967)

23 Controlled clinical study/ (413369)

24 22 or 23 (647900)

25 Random*.tw. (1243699)

26 randomization/ (72965)

For peer review only

27 intermethod comparison/ (235116)

28 placebo.tw. (233779)

29 (compare or compared or comparison).ti. (353942)

30 ((evaluated or evaluate or evaluating or assessed or assess) and (compare or compared or comparing or comparison)).ab. (1742773)

31 (open adj label).tw. (67575)

32 ((double or single or doubly or singly) adj (blind or blinded or blindly)).tw. (166412)

33 double blind procedure/ (131359)

34 parallel group*1.tw. (20690)

35 (crossover or cross over).tw. (76213)

36 ((assign* or match or matched or allocation) adj5 (alternate or group*1 or intervention*1 or patient*1 or subject*1 or participant*1)).tw. (265294)

37 (assigned or allocated).tw. (310200)

38 (controlled adj7 (study or design or trial)).tw. (280066)

39 (volunteer or volunteers).tw. (180358)

40 human experiment/ (307840)

41 trial.ti. (231364)

42 or/25-41 (3776672)

43 42 and 24 (520100)

44 (random* adj sampl* adj7 ("cross section*" or questionnaire*1 or survey* or database*1)).tw. not (comparative study/ or controlled study/ or randomi?ed controlled.tw. or randomly assigned.tw.) (6701)

45 Cross-sectional study/ not (randomized controlled trial/ or controlled clinical study/ or controlled study/ or randomi?ed controlled.tw. or control group*1.tw.) (196889)

46 (((case adj control*) and random*) not randomi?ed controlled).tw. (14459)

47 (Systematic review not (trial or study)).ti. (111407)

48 (nonrandom* not random*).tw. (12428)

49 "Random field*".tw. (1913)

50 (random cluster adj3 sampl*).tw. (1109)

51 (review.ab. and review.pt.) not trial.ti. (671976)

52 "we searched".ab. and (review.ti. or review.pt.) (26759)

53 "update review".ab. (89)

54 (databases adj4 searched).ab. (27931)

For peer review only

55 (rat or rats or mouse or mice or swine or porcine or murine or sheep or lambs or pigs or piglets or rabbit or rabbits or cat or cats or dog or dogs or cattle or bovine or monkey or monkeys or trout or marmoset*1).ti. and animal experiment/ (621221)

56 Animal experiment/ not (human experiment/ or human/) (1278682)

57 or/44-56 (2292431)

58 43 not 57 [Cochrane Embase RTC search filter Jan 2015] (504463)

59 program evaluat*.mp. (15961)

60 health care quality/ (199019)

61 process* evaluat*.mp. (3994)

62 or/59-61 [process evaluation] (216384)

63 21 and 58 and 62 [sedentary behaviour and RCTs and process evaluations] (213)

64 remove duplicates from 63 (209)

For peer review only

PsycINFO (OVID)

1 SEDENTARY BEHAVIOR/ (45)

2 (sedentary or sitting or sedentariness or sedentarism).ti. (7958)

3 ((sedentary or sitting or seated) adj5 (behavio* or lifestyle or life-style)).tw. (11608)

4 ((inactiv* or no exercise or nonexercise or non exercise) adj3 (adult? or men or women or males or females or individuals or people)).tw. (3191)

5 (sedentary adj3 (adult? or men or women or males or females or individuals or people or population?)).tw. (6088)

6 ((sitting or sit or seated or stationary or standing) adj3 (task* or time or bout* or work* or break*)).tw. (6389)

7 low energy expenditure.tw. (196)

8 physical* inactiv*.tw. (10206)

9 (leisure time adj5 (physical* activ* or passive or inactiv*)).tw. (4182)

10 "physical activity level*".tw. (10320)

11 ((sitting or lying) adj2 posture*).tw. (1181)

12 (prolong* adj2 (reclin* or sit or sitting or seated)).tw. (820)

13 chair rise?.tw. (561)

14 "sit* less".tw. (1080)

15 ((light or low) adj "physical activ*").tw. (3123)

16 ((decrease or reduc* or discourag* or lessen*) adj3 (sit or sitting or stand or standing or physical* inactiv*)).tw. (1570)

17 (time adj5 (computer* or television or tv or video game? or videogame? or gaming or screen or media)).tw. (12058)

18 ((watch* or view*) adj5 (television or tv)).tw. (5775)

19 (play* adj5 (video game? or videogame? or computer game?)).tw. (1941)

20 ((computer* or television or tv or video game? or videogame? or gaming) and (sedentary or physical* activity* or sitting or seated or underactiv* or under activ*)).ti. (432)

21 or/1-20 [sedentary behaivour] (68730)

22 Treatment Effectiveness Evaluation/ (0)

23 exp Treatment Outcomes/ (0)

24 Psychotherapeutic Outcomes/ (0)

25 PLACEBO/ (277088)

26 exp Followup Studies/ (0)

For peer review only

27 placebo*.tw. (235252)

28 random*.tw. (1243699)

29 comparative stud*.tw. (77003)

30 (clinical adj3 trial*).tw. (433925)

31 (research adj3 design).tw. (31590)

32 (evaluat* adj3 stud*).tw. (605716)

33 (prospectiv* adj3 stud*).tw. (453513)

34 ((singl* or doubl*or trebl* or tripl*) adj3 (blind* or mask*)).tw. (24389)

35 or/22-34 [RCT filter adapted from Watson RJ, Richardson PH 1999] (2613798)

36 program evaluat*.mp. (15961)

37 process* evaluat*.mp. (3994)

38 evaluation/ (130069)

39 or/36-38 [process evaluation terms] (148923)

40 21 and 35 and 39 [sedentary behaviour and rcts and process evalutions] (267)

For peer review only

Ovid MEDLINE(R)

1 Sedentary Lifestyle/ (7525)

2 (sedentary or sitting or sedentariness or sedentarism).ti. (6452)

3 ((sedentary or sitting or seated) adj5 (behavio* or lifestyle or life-style)).tw. (7249)

4 ((inactiv* or no exercise or nonexercise or non exercise) adj3 (adult? or men or women or males or females or individuals or people)).tw. (2515)

5 (sedentary adj3 (adult? or men or women or males or females or individuals or people or population?)).tw. (4859)

6 ((sitting or sit or seated or stationary or standing) adj3 (task* or time or bout* or work* or break*)).tw. (4603)

7 low energy expenditure.tw. (144)

8 physical* inactiv*.tw. (6591)

9 (leisure time adj5 (physical* activ* or passive or inactiv*)).tw. (3445)

10 "physical activity level*".tw. (6404)

11 ((sitting or lying) adj2 posture*).tw. (998)

12 (prolong* adj2 (reclin* or sit or sitting or seated)).tw. (564)

13 chair rise?.tw. (323)

14 "sit* less".tw. (601)

15 ((light or low) adj "physical activ*").tw. (1853)

16 ((decrease or reduc* or discourag* or lessen*) adj3 (sit or sitting or stand or standing or physical* inactiv*)).tw. (1377)

17 (time adj5 (computer* or television or tv or video game? or videogame? or gaming or screen or media)).tw. (8936)

18 ((watch* or view*) adj5 (television or tv)).tw. (4240)

19 (play* adj5 (video game? or videogame? or computer game?)).tw. (1305)

20 ((computer* or television or tv or video game? or videogame? or gaming) and (sedentary or physical* activity* or sitting or seated or underactiv* or under activ*)).ti. (351)

21 or/1-20 [sedentary behaviour terms] (50991)

22 Program Evaluat*.mp. (62861)

23 "Outcome and Process Assessment (Health Care)"/ (25572)

24 "Process Assessment (Health Care)"/ (4358)

25 process evaluat*.mp. (2608)

26 or/22-25 [process evaluation] (91311)

27 randomized controlled trial.pt. (476630)

28 controlled clinical trial.pt. (92914)

29 randomized.ab. (377791)

30 placebo.ab. (177752)

31 drug therapy.fs. (2086845)

32 randomly.ab. (262246)

33 trial.ab. (392148)

34 groups.ab. (1631334)

35 27 or 28 or 29 or 30 or 31 or 32 or 33 or 34 (4041965)

36 exp animals/ not humans.sh. (4552221)

37 35 not 36 [Cochrane RCT filter 2008, sensitivity maximimising] (3448772)

38 21 and 26 and 37 [sedentary behaviour and process evaluation and RCTs] (420)

For peer review only

OVID MEDLINE(R) and Epub Ahead of Print, In-Process & Other Non-Indexed Citations

1 Sedentary Lifestyle/ (7594)

2 (sedentary or sitting or sedentariness or sedentarism).ti. (7865)

3 ((sedentary or sitting or seated) adj5 (behavio* or lifestyle or life-style)).tw. (8993)

4 ((inactiv* or no exercise or nonexercise or non exercise) adj3 (adult? or men or women or males or females or individuals or people)).tw. (2927)

5 (sedentary adj3 (adult? or men or women or males or females or individuals or people or population?)).tw. (5551)

6 ((sitting or sit or seated or stationary or standing) adj3 (task* or time or bout* or work* or break*)).tw. (5742)

7 low energy expenditure.tw. (166)

8 physical* inactiv*.tw. (7962)

9 (leisure time adj5 (physical* activ* or passive or inactiv*)).tw. (3859)

10 "physical activity level*".tw. (7765)

11 ((sitting or lying) adj2 posture*).tw. (1165)

12 (prolong* adj2 (reclin* or sit or sitting or seated)).tw. (721)

13 chair rise?.tw. (374)

14 "sit* less".tw. (693)

15 ((light or low) adj "physical activ*").tw. (2305)

16 ((decrease or reduc* or discourag* or lessen*) adj3 (sit or sitting or stand or standing or physical* inactiv*)).tw. (1670)

17 (time adj5 (computer* or television or tv or video game? or videogame? or gaming or screen or media)).tw. (10911)

18 ((watch* or view*) adj5 (television or tv)).tw. (4851)

19 (play* adj5 (video game? or videogame? or computer game?)).tw. (1650)

20 ((computer* or television or tv or video game? or videogame? or gaming) and (sedentary or physical* activity* or sitting or seated or underactiv* or under activ*)).ti. (415)

21 or/1-20 [sedentary behaviour terms] (60730)

22 Program Evaluat*.mp. (62861)

23 "Outcome and Process Assessment (Health Care)"/ (25610)

24 "Process Assessment (Health Care)"/ (4370)

25 process evaluat*.mp. (3311)

26 or/22-25 [process evaluation] (93381)

For peer review only

27 randomized controlled trial.pt. (477874)

28 controlled clinical trial.pt. (92968)

29 randomized.ab. (437254)

30 placebo.ab. (196103)

31 drug therapy.fs. (2090621)

32 randomly.ab. (307192)

33 trial.ab. (456911)

34 groups.ab. (1890503)

35 27 or 28 or 29 or 30 or 31 or 32 or 33 or 34 (4396098)

36 exp animals/ not humans.sh. (4557181)

37 35 not 36 [Cochrane RCT filter 2008, sensitivity maximimising] (3801789)

38 21 and 26 and 37 [sedentary behaviour and process evaluation and RCTs] (449)

For peer review only

Web of Science (Clarivate)

Set	Results	Save History / Create Alert · Open Saved History	Sets	AND OR Combine	Select All · Delete
# 23	121	#22 AND #21 AND #18<br>*Indexes=SCI-EXPANDED, SSCI, CPCI-S, CPCI-SSH Timespan=1900-2019*	Edit	☐	☐
# 22	149,472	#1 or #2 or #3 or #4 or #5 or #6 or #7 or #8 or #9 or #10 or #11 or #12 or #13 or #14 or #15 or #16 or #17<br>*Indexes=SCI-EXPANDED, SSCI, CPCI-S, CPCI-SSH Timespan=1900-2019*	Edit	☐	☐
# 21	13,136	#20 OR #19<br>*Indexes=SCI-EXPANDED, SSCI, CPCI-S, CPCI-SSH Timespan=1900-2019*	Edit	☐	☐
# 20	4,451	TS=("process evaluat*")<br>*Indexes=SCI-EXPANDED, SSCI, CPCI-S, CPCI-SSH Timespan=1900-2019*	Edit	☐	☐
# 19	8,811	TS=("program* evaluat*")<br>*Indexes=SCI-EXPANDED, SSCI, CPCI-S, CPCI-SSH Timespan=1900-2019*	Edit	☐	☐
# 18	2,014,426	TOPIC: (random* or RCT or placebo or clinical Near/1 trial*)<br>*Indexes=SCI-EXPANDED, SSCI, CPCI-S, CPCI-SSH Timespan=1900-2019*	Edit	☐	☐
# 17	759	TI=((computer* or television or tv or "video game?" or videogame? or gaming) and (sedentary or "physical* activity*" or sitting or seated or underactiv* or under activ*))<br>*Indexes=SCI-EXPANDED, SSCI, CPCI-S, CPCI-SSH Timespan=1900-2019*	Edit	☐	☐
# 16	3,591	TS= (play* NEAR/5 ("video game*" or "videogame*" or "computer game*"))<br>*Indexes=SCI-EXPANDED, SSCI, CPCI-S, CPCI-SSH Timespan=1900-2019*	Edit	☐	☐
# 15	8,648	TS=((watch* or view*) NEAR/5 (television or tv))<br>*Indexes=SCI-EXPANDED, SSCI, CPCI-S, CPCI-SSH Timespan=1900-2019*	Edit	☐	☐
# 14	62,624	TS=(time NEAR/5 (computer* or television or tv or "video game*" or videogame* or gaming or screen or media))<br>*Indexes=SCI-EXPANDED, SSCI, CPCI-S, CPCI-SSH Timespan=1900-2019*	Edit	☐	☐
# 13	6,328	TS=((decrease or reduc* or discourag* or lessen*) NEAR/3((sit or sitting or stand or standing or "physical* inactiv*")))<br>*Indexes=SCI-EXPANDED, SSCI, CPCI-S, CPCI-SSH Timespan=1900-2019*	Edit	☐	☐
# 12	3,932	TOPIC: (((light or low) near/1 "physical activ*"))<br>*Indexes=SCI-EXPANDED, SSCI, CPCI-S, CPCI-SSH Timespan=1900-2019*	Edit	☐	☐
# 11	978	TS=("sit* less")<br>*Indexes=SCI-EXPANDED, SSCI, CPCI-S, CPCI-SSH Timespan=1900-2019*	Edit	☐	☐
# 10	21,895	TS=((sitting or sit or seated or stationary or standing) NEAR/3 (task* or time or bout* or work* or break*))<br>*Indexes=SCI-EXPANDED, SSCI, CPCI-S, CPCI-SSH Timespan=1900-2019*	Edit	☐	☐
# 9	380	TOPIC: ("chair rise")<br>*Indexes=SCI-EXPANDED, SSCI, CPCI-S, CPCI-SSH Timespan=1900-2019*	Edit	☐	☐
# 8	3,681	TOPIC: ((nonexercis* or "non exercis*" or "no exercis*"))<br>*Indexes=SCI-EXPANDED, SSCI, CPCI-S, CPCI-SSH Timespan=1900-2019*	Edit	☐	☐
# 7	1,800	TOPIC: (((sitting or lying) near/2 posture*))<br>*Indexes=SCI-EXPANDED, SSCI, CPCI-S, CPCI-SSH Timespan=1900-2019*	Edit	☐	☐
# 6	15,258	TS=("physical activity level*" or "physical* inactiv*")<br>*Indexes=SCI-EXPANDED, SSCI, CPCI-S, CPCI-SSH Timespan=1900-2019*	Edit	☐	☐
# 5	3,838	TS=(("leisure time" NEAR/5 ("physical* activ*" or passive or inactiv*)))<br>*Indexes=SCI-EXPANDED, SSCI, CPCI-S, CPCI-SSH Timespan=1900-2019*	Edit	☐	☐
# 4	7,443	TS=((sedentary) near/3 (adult* or men or women or males or females or individuals or people or population*))<br>*Indexes=SCI-EXPANDED, SSCI, CPCI-S, CPCI-SSH Timespan=1900-2019*	Edit	☐	☐
# 3	2,596	TS=((inactive* or "non exercise" or "nonexercise" or "no exercise") near/3 (adult* or men or women or males or females or individuals or people))<br>*Indexes=SCI-EXPANDED, SSCI, CPCI-S, CPCI-SSH Timespan=1900-2019*	Edit	☐	☐
# 2	13,139	TS=(((sedentary or sitting or seated) NEAR/5 (behavio* or lifestyle or life-style)))<br>*Indexes=SCI-EXPANDED, SSCI, CPCI-S, CPCI-SSH Timespan=1900-2019*	Edit	☐	☐
# 1	16,294	TI=((sedentary or sitting or sedentariness or sedentarism))<br>*Indexes=SCI-EXPANDED, SSCI, CPCI-S, CPCI-SSH Timespan=1900-2019*	Edit	☐	☐

ProQuest Dissertations & Theses A&I

Databases:

ProQuest Dissertations & Theses A&I 21.3.19

Select item 1	**S1**	ti((computer* OR television OR tv OR "video game" OR "videogame*" OR gaming) AND (sedentary OR physical* activity* OR sitting OR seated OR underactiv* OR under activ*)) OR ti(sedentary OR sitting OR elementariness OR sedentary OR (sedentary OR sitting OR seated) N5 (behavio* OR lifestyle OR life-style)) OR ti((sitting OR sit OR seated OR stationary OR standing) N3 (task* OR time OR bout* OR work* OR break*)) OR ti("physical* inactiv*" OR "chair rise*" OR "low energy expenditure" OR "sit less") OR ti((watch* OR view*) N5 (television OR tv)) OR ti(play* N5 ("video game*" OR videogame* OR "computer game*")) OR ti(time N5 (computer* OR television OR tv OR "video game*" OR videogame* OR gaming OR screen OR media)) OR ti((computer* OR television OR tv OR "video game" OR "videogame*" OR gaming) AND (sedentary OR physical* activity* OR sitting OR seated OR underactiv* OR "under activ*")) AND ti("process* evaluation*" OR "program* evaluation*") AND ti(Random* OR RCT OR clinical N1 trial*)	ProQuest Dissertations & Theses A&I	**699**

For peer review only

BMJ Open

A protocol for a systematic review of process evaluations of interventions investigating sedentary behaviour in adults

Journal:	*BMJ Open*
Manuscript ID	bmjopen-2019-031291
Article Type:	Protocol
Date Submitted by the Author:	26-Apr-2019
Complete List of Authors:	Corepal, Rekesh; Bradford Royal Infirmary, Academic Unit of Elderly Care and Rehabilitation<br>Hall, Jessica; Bradford Royal Infirmary, Academic Unit of Elderly Care and Rehabilitation<br>Birch, Karen; University of Leeds Faculty of Biological Sciences<br>Carter, Gill; Bradford Institute for Health Research, Academic Unit of Elderly Care and Rehabilitation<br>English, Coralie; University of Newcastle, School of Health Sciences<br>Farrin, Amanda; University of Leeds, Clinical Trials Research Unit<br>Fitzsimons, Claire F.; University of Edinburgh, ISPEHS<br>Forster, Anne; University of Leeds, Academic Unit of Elderly Care and Rehabilitation<br>Hall, Jennifer; Bradford Teaching Hospitals NHS Foundation Trust, Academic Unit of Elderly Care and Rehabilitation<br>Holloway, Ivana; University of Leeds, Clinical Trials Research Unit<br>Lawton, Rebecca; University of Leeds, Institute of Psychological Sciences; Bradford Institute for Health Research, Quality and Safety Research<br>Mead, Gillian; University of Edinburgh, Geriatric Medicine<br>Morton, Sarah; University of Edinburgh, Geriatric Medicine<br>Patel, Anita; Queen Mary University of London,<br>Clarke, David; University of Leeds, Academic Unit of Elderly Care and Rehabilitation
Keywords:	Systematic review, Process evaluation, Sedentary behaviour, Protocol

SCHOLARONE™
Manuscripts

Title page

A protocol for a systematic review of process evaluations of interventions investigating sedentary behaviour in adults

Authors:

[1]Rekesh Corepal Rekesh.Corepal@bthft.nhs.uk(corresponding author); [1]Jessica Hall Jessica.Hall@bthft.nhs.uk; [2]Karen Birch K.M.Birch@leeds.ac.uk; [1]Gill Carter GillCarter54@yahoo.co.uk; [3]Coralie English Coralie.English@newcastle.edu.au; [4]Amanda Farrin A.J.Farrin@leeds.ac.uk; [5]Claire Fitzsimons Claire.Fitzsimons@ed.ac.uk; [1]Anne Forster A.Forster@Leeds.ac.uk; [1]Jennifer Hall Jennifer.Hall@bthft.nhs.uk; [4]Ivana Holloway I.Holloway@leeds.ac.uk; [6]Rebecca Lawton R.J.Lawton@leeds.ac.uk; [7]Gillian Mead Gillian.E.Mead@ed.ac.uk; [7]Sarah Morton Sarah.Morton@ed.ac.uk; Anita Patel AnitaPatelConsulting@gmail.com; [1]David Clarke D.J.Clarke@leeds.ac.uk.

[1]Academic Unit of Elderly Care and Rehabilitation, Bradford Institute for Health Research, Temple Bank House, Bradford Royal Infirmary, Bradford, BD9 6RJ

[2]Garstang 5.76, Faculty of Biological Sciences, University of Leeds. Leeds, LS2 9JT

[3]Physiotherapy Program, School of Health Sciences, Faculty of Health and Medicine, HC-36 Hunter Building, The University of Newcastle, Australia, NSW 2308

[4]Leeds Institute of Clinical Trials Research, Worsley Building, Clarendon Way, University of Leeds, Leeds, LS2 9NL

[5]Institute for Sport, Physical Education and Health, The University of Edinburgh, St Leonard's land, Holyrood Road, Edinburgh, EH8 8AQ

[6]Yorkshire Quality and Safety, Bradford Institute for Health Research, Temple Bank House, Bradford Royal Infirmary, Bradford, BD9 6RJ

[7]Geriatric Medicine, Division of Health Sciences, Centre for Clinical Brain Sciences, Room F1424, Royal Infirmary, Edinburgh, EH16 4SB

Word count: 1915

For peer review only - http://bmjopen.bmj.com/site/about/guidelines.xhtml

Abstract

Introduction

Sedentary behaviour is defined as any waking behaviour characterised by low energy expenditure ≤1.5 Metabolic Equivalents (METs) while in a sitting, lying or reclining posture. The rapidly expanding evidence base suggests that sedentary behaviour has a detrimental effect on health, wellbeing, and is associated with all-cause mortality.

We aim to review process evaluations of randomised controlled trials (RCTs) which include a measure of sedentary behaviour in adults in order to develop an understanding of intervention content, mechanisms of impact, implementation and delivery approaches and contexts, in which interventions were reported to be effective or ineffective. A secondary aim is to explore participants, family, and staff experiences of such interventions.

Methods and analysis:

Nine electronic databases, websites of relevant organisations, and included studies from previous similar reviews will be searched. No language restrictions will be applied. Eligible studies will be process evaluations of RCTs which include a measure of sedentary behaviour in adults. Studies with interventions delivered in schools, colleges, universities or the workplace will be excluded. Two reviewers will perform study selection, data extraction, and quality assessment. Disagreements between reviewers will be resolved by a consensus-based discussion or a third reviewer. Process evaluation data to be extracted include: the aims and methods used in the process evaluation; implementation data; mechanisms of impact; contextual factors; participant, family, and staff experiences of the interventions. Trial data to be extracted include: trial design; participant numbers; duration and content of the intervention; primary and secondary outcome results if available in the process evaluation paper.

For peer review only - http://bmjopen.bmj.com/site/about/guidelines.xhtml

A narrative approach will be adopted to synthesise and report qualitative and quantitative data. Reporting of the review will be informed by Preferred Reporting Items for Systematic Review and Meta-analysis (PRISMA) guidance.

Ethics and dissemination:

Ethical approval is not required as it is a protocol for a systematic review. Review findings will be disseminated through peer-reviewed publications and conference presentations.

Protocol registration number: CRD42018087403

Keywords: Sedentary, Adults, Process evaluation, Systematic review, Protocol

Article Summary

Strengths and limitations of this study

- This systematic review protocol follows the Preferred Reporting Items for Systematic reviews and Meta-Analyses (PRISMA-P) guidelines
- This systematic review addresses a gap in the current evidence-base by providing a comprehensive assessment of the implementation, mechanisms of impact, and contextual factors which may influence the effectiveness of RCTs investigating sedentary behaviour in adults
- No country, language, or time restrictions will be applied to the search strategy
- The findings from this systematic review will contribute to the development of future interventions designed to reduce sedentary behaviour.

For peer review only - http://bmjopen.bmj.com/site/about/guidelines.xhtml

BACKGROUND

Outcome evaluations, such as randomised controlled trials (RCTs), are important to understanding intervention effectiveness, however, in isolation, they fail to account for how interventions function, why they are successful or not, and for whom [1]. Process evaluations can help to provide this necessary insight [2]. Undertaken alongside outcome evaluations, they include quantitative, qualitative, or mixed-methods approaches *"which aim to understand the functioning of an intervention, by examining implementation, mechanisms of impact and contextual factors"* [3, pg 8].

Process evaluations may also explore the theoretical and logic models informing or underpinning interventions. A theoretical model may be used by researchers in the development of complex interventions to identify key concepts of interest which may be influential in bringing about a desired outcome or change. Logic models are one method of making theoretical assumptions clear, as they graphically illustrate the link between expected outcomes and intervention activities/processes designed to bring about these outcomes [3 4].

Rationale

Sedentary behaviour is defined as any waking behaviour characterised by low energy expenditure ≤1.5 Metabolic Equivalents (METs) while in a sitting, lying or reclining posture [5]. It has emerged as an important public health issue in the last two decades and has become the focus of considerable clinical, policy and practice research as evidence supporting the detrimental effects of sedentary behaviour on health and wellbeing has increased [6-8].

The negative impact of sedentary behaviour has been highlighted for a number of parameters related to health [8 9], including reduced physical function [10 11], increased symptoms of depression [12], anxiety [13] and cardiovascular risk [14 15]. The evidence reporting the deleterious associations with sedentary behaviour on cardiovascular risk in adults is strong. An overview of reviews concluded that sedentary behaviour is associated with cardiovascular mortality regardless of the level of physical activity, and recommended that further interventional research be undertaken [8].

For peer review only - http://bmjopen.bmj.com/site/about/guidelines.xhtml

The effectiveness of interventions to reduce sedentary behaviour has been synthesised in systematic reviews and meta-analyses. [16-18]. However, such work often fails to provide a detailed understanding of the functioning of the interventions [19]. This systematic review of process evaluations aims to fill this gap in the literature, and contribute to the development of future interventions designed to reduce sedentary behaviour.

Aims and objectives

Review aim:

To identify and review previously conducted process evaluations of interventions investigating sedentary behaviour in adults, in order to develop an understanding of intervention content, mechanisms of impact, implementation and delivery approaches and contexts, in which interventions were reported to be effective or ineffective. A secondary aim is to explore participants, family, and staffs experiences of such interventions.

Objectives:

i. To examine the trial data (e.g. design of the intervention, participants numbers, duration and content of the intervention, and primary and secondary outcome results if available in the process evaluation paper).
ii. Establish whether a logic model or a theoretical model was used to explain how the intervention was intended to work.
iii. Establish whether the intervention was delivered as intended.
iv. Explore intended or unintended mechanisms of action reported to influence the effectiveness of interventions.
v. Understand the barriers and facilitators to delivery of, and participation in, the intervention and any recommendations made to address such barriers and facilitators.
vi. To examine the qualitative data concerning the understanding and experiences of the intervention from the perspectives of participants, family/carers, and staff.

For peer review only - http://bmjopen.bmj.com/site/about/guidelines.xhtml

Qualitative data related to exploring perceptions, views, and lived experiences of sedentary behaviour, but not related to receipt or delivery of the intervention will be transferred to a concurrent qualitative systematic review (Prospero registration number: CRD42017083436).

METHODS AND ANALYSIS

This protocol has been developed following the Preferred Reporting Items for Systematic Review and Meta-analysis Protocols (PRISMA-P) guidelines [20], as shown in the PRISMA-P checklist (see Additional file 1). The systematic review is prospectively registered with PROSPERO (Prospective Register of Systematic Reviews). Reporting of the systematic review will be informed by Preferred Reporting Items for Systematic Review and Meta-analysis (PRISMA) guidance [21]. Important amendments made to the protocol will be documented and published alongside the results of the systematic review.

Methodological considerations associated with this review

Inclusion and exclusion criteria

Types of studies**:** Studies that are explicitly identified as a process evaluation, or studies that aim to understand the functioning of an intervention by examining implementation, mechanisms of impact, and contextual factors [3, pg 8]. (E.g. implementation processes, patient and staff barriers and facilitators, participants' experiences of delivery or receipt of the intervention).

We will include studies that are conducted alongside RCTs which include a measure of sedentary behaviour in adults. Feasibility RCTs will be included provided there is random allocation. Cohort and before and after studies will be excluded.

Types of Participants: All studies involving adults, including both a clinical and non-clinical population. The definition for being an 'adult' will be whichever is used in individual papers; for example, if a paper defines an adult as someone over 16 years, then this paper will be included even if other papers define adulthood differently. Studies which include ≥10% children or adolescent participants will be excluded.

For peer review only

Interventions: Any study which includes a measure of sedentary behaviour will be included even if reducing sedentary behaviour is not the primary outcome (e.g. physical activity or weight management interventions).

Interventions that are delivered primarily in schools, colleges, universities or the workplace, and papers that do not report any measures of sitting time or sedentary behaviour as an outcome measure will be excluded. Studies where the main aim is to investigate the acute (immediate) effects of breaking up sitting time as part of a supervised (usually laboratory based) intervention will also be excluded.

Comparators: In the source trial, the intervention group may be compared to:

- No active treatment
- Usual care
- Attention controls
- Waitlist controls
- Alternative treatments

Where process evaluations include data from control groups, these data will be extracted.

Information sources

Electronic searches

In collaboration with information specialist colleagues, informed by guidance from Booth [22], comprehensive search strategies will be developed using controlled vocabulary and free text terms. Date and/or language restrictions will not be applied in this review.

We will search the following electronic databases:

- MEDLINE
- Medline in Process & Other Non-Indexed Citations

For peer review only

For peer review only - http://bmjopen.bmj.com/site/about/guidelines.xhtml

- Allied and Complementary Medicine Database (AMED)EMBASE
- Cochrane Database of Systematic Reviews.
- CINAHL (Cumulative Index to Nursing and Allied Health Literature)
- PsycINFO
- SportDiscus
- Cochrane Central Register of Controlled Trials
- Web of Science
- Dissertation Abstracts

Searching other relevant sources

In addition to the electronic database searches, we will identify process evaluations through existing systematic reviews of interventions studies. This will include a number of steps:

- Examining the RCTs reported within the existing reviews to determine whether they meet the inclusion criteria.
- Reading the RCT publications to identify any process evaluation work. If a process evaluation is referred to, but no data is reported in these publications, we will:
    i. Match the RCTs to any process evaluations identified through the electronic searches (above).
    ii. If they cannot be matched, we will identify linked published process evaluations by performing citation searching (Google Scholar, PubMed, and Web of Science) and also contact authors of the trial publications to request information on any published or unpublished process evaluations.

A draft MEDLINE search strategy is included in Additional file 2. Following any necessary refinements, the search strategy will be adapted for use with the other

For peer review only - http://bmjopen.bmj.com/site/about/guidelines.xhtml

For peer review only

electronic databases.

Study records

Data management

We will download references identified in searches (electronic database and additional searches) into Endnote X7 reference management software. Once duplicates are removed, the remaining references will be exported into Covidence (www.covidence.org); an online systematic review tool recommended by the Cochrane Collaboration.

Selection process

The screening process will be undertaken using Covidence. Two review authors will independently assess the titles and abstracts of records and exclude obviously irrelevant papers. We will obtain the full text of the remaining papers, and two authors will assess the papers against the inclusion criteria for the review to determine their eligibility for inclusion. Foreign language papers will be translated. The review authors will resolve disagreements through a consensus-based decision, or if necessary, discussion with a third reviewer.

Data extraction process

Two review authors will independently extract and record data from included studies using a standardised data extraction form. The data extraction form will be guided by the Medical Research Council (MRC) guidance for process evaluations [1], and previous research which has identified key components for conducting and reporting process evaluations [23 24]. Reviewers will pilot the data extraction form with a sample of included papers and amendments will be made as necessary. After piloting, data extraction will be completed using Covidence. Study authors will be contacted if additional information is required. Following data extraction two reviewers will aim to resolve any discrepancies by a consensus-based decision, or if necessary, discussion with a third reviewer.

For peer review only - http://bmjopen.bmj.com/site/about/guidelines.xhtml

We will extract data about the RCT and the process evaluation. Data to be extracted includes:

1. The trial design and trial information:

    a) The number of participants randomised to each group, and demographic information.

    b) The duration and content of what is provided to the intervention group and the comparator group.

    c) Primary and secondary outcome results including adverse events measured at post-intervention and follow up.

2. The aims and objectives of the process evaluation and whether the process evaluation was pre-specified or post-hoc.

3. The methods used to conduct the process evaluation.

4. The number of sites sampled for the process evaluation, and sample characteristics (e.g. recruitment and maintenance of participants or participating sites, reach of the intervention into the target population, age, and gender).

5. Implementation data (e.g. what is intended to be delivered? How is delivery achieved? What is delivered? How is adherence measured?

6. Mechanisms of impact (drawing on the logic model or intervention theory used, identified mediators of change, and responses to and interactions with the intervention).

7. Contextual factors that influence implementation, intervention mechanisms, and outcomes.

8. Participants, family/carers, and staff views and experiences of the interventions, including barriers and facilitators. Experiences of control group participants relating to their involvement in the trial.

9. Any conflicts of interest declared by the authors.

For peer review only - http://bmjopen.bmj.com/site/about/guidelines.xhtml

Outcomes and prioritisation

To meet our research aims and objectives, the outcomes of interest for this study include the following:

1. The outcome results from the intervention.
2. Findings from the process data relating to implementation (intended delivery and fidelity to the intervention plan).
3. Adherence to the intervention and how this is measured.
4. Intended and unintended mechanisms of impact.
5. Barriers and facilitators to delivery or participation in the intervention.
6. Adaptations made to improve delivery of the intervention.
7. All participants experiences of the intervention (delivery and receipt).

Findings will clarify key factors that affect intervention delivery and participation. This will provide contextual information useful for explaining why interventions were effective or ineffective, and how interventions could be refined.

Quality assessment

Currently, there is no quality assessment tool designed for judging the quality of process evaluations. Process evaluations can incorporate a combination of both qualitative and quantitative data. Therefore, methodological quality will be evaluated using the Mixed Methods Appraisal Tool (MMAT), which is designed to concurrently assess qualitative, quantitative, and mixed methods studies [25]. Assessment of reporting quality will be guided by Grant et al's framework for reporting process evaluations of cluster randomised controlled trials [24]. Two reviewers will independently assess each study and discrepancies will be resolved by a third reviewer. We will not exclude studies based on findings from the quality assessment.

For peer review only - http://bmjopen.bmj.com/site/about/guidelines.xhtml

Data synthesis

Narrative approach to synthesising data

In this review, we will undertake a narrative approach to synthesising data. The synthesis will provide detailed written commentary on the data extracted in accordance with the factors outlined in the 'data collection process' section. This will advance our understandings of the intervention context, its delivery, and the mechanisms reported to be effective or ineffective.

Patient and public involvement

As this research will be based on secondary data, there will be no patient and public involvement in the design, interpretation, or dissemination of the findings.

DISCUSSION

This systematic review aims to review process evaluations of randomised controlled trials which include a measure of sedentary behaviour in adults, in order to develop an understanding of intervention content, mechanisms of impact, implementation and delivery approaches and contexts, in which interventions were reported to be effective or ineffective. A secondary aim is to explore participants, family, and staffs experiences of such interventions. An additional aim is to explore participants, family/carers, and staff understanding and experiences of such interventions. The findings from this systematic review will contribute to the development of future interventions designed to reduce sedentary behaviour.

For peer review only - http://bmjopen.bmj.com/site/about/guidelines.xhtml

List of abbreviations

AMED: Allied and Complementary Medicine Database

CINAHL: Cumulative Index to Nursing and Allied Health Literature

METS: Metabolic Equivalents

MMAT: Mixed Methods Appraisal Tool

MRC: Medical Research Council

NIHR: National Institute for Health Research

PRISMA: Preferred Reporting Items for Systematic Reviews and Meta-Analyses

PRISMA-P: Preferred Reporting Items for Systematic Review and Meta-analysis Protocols

PROSPERO: Prospective Register of Systematic Reviews

RCT: Randomised Controlled Trial

Author Contributions

Plans for this systematic review were conceived by the Programme Management Group of the RECREATE Programme (AF,KB,GC,CE,AF,CF,IH,RL,GM,AP,DC). This protocol was initially drafted by DC, RC, JH, and AF. Subsequent drafts were commented on and revised by all members of the PMG. All authors have approved submission.

Competing interests

The authors declare that they have no competing interests.

Acknowledgements

We acknowledge the help and support of our Information Scientist, Deirdre Andre, University of Leeds.

We are grateful for the funding provided by the National Institute for Health Research (NIHR).

For peer review only - http://bmjopen.bmj.com/site/about/guidelines.xhtml

Availability of data

Not applicable.

Funding statement

This report presents independent research funded by the National Institute for Health Research (NIHR) under its Programme Grants for Applied Research Programme (Development and evaluation of strategies to reduce sedentary behaviour in patients after stroke and improve outcomes, Reference number RP-PG-0615-20019). The views expressed are those of the author(s) and not necessarily those of the NHS, the NIHR or the Department of Health.

peer review only

References

1. Moore GF, Audrey S, Barker M, et al. Process evaluation of complex interventions: Medical Research Council guidance. *BMJ* 2015;350:h1258. doi: 10.1136/bmj.h1258

2. Craig P, Dieppe P, Macintyre S, et al. Developing and evaluating complex interventions: the new Medical Research Council guidance. *BMJ* 2008;337:a1655. doi: 10.1136/bmj.a1655

3. Moore GF, Audrey S, Barker M, et al. Process evlauations of complex interventions: UK Medical Research Council (MRC) guidance. London: Population Health Sciences Research Network, 2014.

4. Kellogg Foundation W. Using logic models to bring together planning, evaluation, and action: Logic model development guide. *Battle Creek, Mich: WK Kellogg Foundation* 2004

5. Tremblay MS, Aubert S, Barnes JD, et al. Sedentary Behavior Research Network (SBRN) - Terminology Consensus Project process and outcome. *Int J Behav Nutr Phys Act* 2017;14(1):75. doi: 10.1186/s12966-017-0525-8

6. Healy GN, Dunstan DW, Salmon J, et al. Breaks in sedentary time: beneficial associations with metabolic risk. *Diabetes Care* 2008;31(4):661-6. doi: 10.2337/dc07-2046

7. Owen N, Healy GN, Matthews CE, et al. Too much sitting: the population health science of sedentary behavior. *Exerc Sport Sci Rev* 2010;38(3):105-13. doi: 10.1097/JES.0b013e3181e373a2

8. de Rezende LF, Rey-Lopez JP, Matsudo VK, et al. Sedentary behavior and health outcomes among older adults: a systematic review. *BMC Public Health* 2014;14:333. doi: 10.1186/1471-2458-14-333

9. Wullems JA, Verschueren SM, Degens H, et al. A review of the assessment and prevalence of sedentarism in older adults, its physiology/health impact and non-exercise mobility counter-measures. *Biogerontology* 2016;17(3):547-65. doi: 10.1007/s10522-016-9640-1

10. Gennuso KP, Gangnon RE, Matthews CE, et al. Sedentary behavior, physical activity, and markers of health in older adults. *Med Sci Sports Exerc* 2013;45(8):1493-500. doi: 10.1249/MSS.0b013e318288a1e5

11. Santos DA, Silva AM, Baptista F, et al. Sedentary behavior and physical activity are independently related to functional fitness in older adults. *Exp Gerontol* 2012;47(12):908-12. doi: 10.1016/j.exger.2012.07.011

12. Teychenne M, Ball K, Salmon J. Sedentary behavior and depression among adults: a review. *Int J Behav Med* 2010;17(4):246-54. doi: 10.1007/s12529-010-9075-z

For peer review only - http://bmjopen.bmj.com/site/about/guidelines.xhtml

13. Teychenne M, Costigan SA, Parker K. The association between sedentary behaviour and risk of anxiety: a systematic review. *BMC Public Health* 2015;15:513. doi: 10.1186/s12889-015-1843-x

14. Edwardson CL, Gorely T, Davies MJ, et al. Association of sedentary behaviour with metabolic syndrome: a meta-analysis. *PLoS One* 2012;7(4):e34916. doi: 10.1371/journal.pone.0034916

15. Same RV, Feldman DI, Shah N, et al. Relationship Between Sedentary Behavior and Cardiovascular Risk. *Curr Cardiol Rep* 2016;18(1):6. doi: 10.1007/s11886-015-0678-5

16. Shrestha N, Grgic J, Wiesner G, et al. Effectiveness of interventions for reducing non-occupational sedentary behaviour in adults and older adults: a systematic review and meta-analysis. *Br J Sports Med* 2018 doi: 10.1136/bjsports-2017-098270

17. Martin A, Fitzsimons C, Jepson R, et al. Interventions with potential to reduce sedentary time in adults: systematic review and meta-analysis. *Br J Sports Med* 2015;49(16):1056-63. doi: 10.1136/bjsports-2014-094524

18. Thraen-Borowski KM, Ellingson LD, Meyer JD, et al. Nonworksite Interventions to Reduce Sedentary Behavior among Adults: A Systematic Review. *Transl J Am Coll Sports Med* 2017;2(12):68-78. doi: 10.1249/TJX.0000000000000036

19. Linnan L, Steckler A. Process evaluation for public health interventions and research: Jossey-Bass 2002.

20. Moher D, Shamseer L, Clarke M, et al. Preferred reporting items for systematic review and meta-analysis protocols (PRISMA-P) 2015 statement. *Syst Rev* 2015;4:1. doi: 10.1186/2046-4053-4-1

21. Moher D, Liberati A, Tetzlaff J, et al. Preferred reporting items for systematic reviews and meta-analyses: the PRISMA statement. *BMJ* 2009;339:b2535. doi: 10.1136/bmj.b2535

22. Booth A. Searching for studies. In: Noyes J, Booth A, Hannes K, et al., eds. Supplementary Guidance for Inclusion of Qualitative Research in Cochrane Systematic Reviews of Interventions: Cochrane Collaboration Qualitative Methods Group, 2011.

23. Baranowski T, Stables G. Process evaluations of the 5-a-day projects. *Health Educ Behav* 2000;27(2):157-66. doi: 10.1177/109019810002700202

24. Grant A, Treweek S, Dreischulte T, et al. Process evaluations for cluster-randomised trials of complex interventions: a proposed framework for design and reporting. *Trials* 2013;14:15. doi: 10.1186/1745-6215-14-15

For peer review only

For peer review only - http://bmjopen.bmj.com/site/about/guidelines.xhtml

25. Pace R, Pluye P, Bartlett G, et al. Testing the reliability and efficiency of the pilot Mixed Methods Appraisal Tool (MMAT) for systematic mixed studies review. *Int J Nurs Stud* 2012;49(1):47-53. doi: 10.1016/j.ijnurstu.2011.07.002

For peer review only

For peer review only - http://bmjopen.bmj.com/site/about/guidelines.xhtml

Additional file 1: PRISMA –P checklist

PRISMA-P (Preferred Reporting Items for Systematic review and Meta-Analysis Protocols) 2015 checklist: recommended items to address in a systematic review protocol*

Section and topic	Item #	Checklist item	Page #
**ADMINISTRATIVE INFORMATION**			
Title:			
Identification	1a	Identify the report as a protocol of a systematic review	1,6
Update	1b	If the protocol is for an update of a previous systematic review, identify as such	N/A*
Registration	2	If registered, provide the name of the registry (such as PROSPERO) and registration number	3
Authors:			
Contact	3a	Provide name, institutional affiliation, e-mail address of all protocol authors; provide physical mailing address of corresponding author	1
Contributions	3b	Describe contributions of protocol authors and identify the guarantor of the review	14
Amendments	4	If the protocol represents an amendment of a previously completed or published protocol, identify as such and list changes; otherwise, state plan for documenting important protocol amendments	6
Support:			
Sources	5a	Indicate sources of financial or other support for the review	14
Sponsor	5b	Provide name for the review funder and/or sponsor	14
Role of sponsor or funder	5c	Describe roles of funder(s), sponsor(s), and/or institution(s), if any, in developing the protocol	14

For peer review only

**INTRODUCTION**			
Rationale	6	Describe the rationale for the review in the context of what is already known	4
Objectives	7	Provide an explicit statement of the question(s) the review will address with reference to participants, interventions, comparators, and outcomes (PICO)	5
**METHODS**			
Eligibility criteria	8	Specify the study characteristics (such as PICO, study design, setting, time frame) and report characteristics (such as years considered, language, publication status) to be used as criteria for eligibility for the review	6-7
Information sources	9	Describe all intended information sources (such as electronic databases, contact with study authors, trial registers or other grey literature sources) with planned dates of coverage	7-8
Search strategy	10	Present draft of search strategy to be used for at least one electronic database, including planned limits, such that it could be repeated	8 Additional file 2
Study records:			
Data management	11a	Describe the mechanism(s) that will be used to manage records and data throughout the review	9
Selection process	11b	State the process that will be used for selecting studies (such as two independent reviewers) through each phase of the review (that is, screening, eligibility and inclusion in meta-analysis)	9
Data collection process	11c	Describe planned method of extracting data from reports (such as piloting forms, done independently, in duplicate), any processes for obtaining and confirming data from investigators	9
Data items	12	List and define all variables for which data will be sought (such as PICO items, funding sources), any pre-planned data assumptions and simplifications	10-11
Outcomes and prioritization	13	List and define all outcomes for which data will be sought, including prioritization of main and additional outcomes, with rationale	10-11

For peer review only

Risk of bias in individual studies	14	Describe anticipated methods for assessing risk of bias of individual studies, including whether this will be done at the outcome or study level, or both; state how this information will be used in data synthesis	11
Data synthesis	15a	Describe criteria under which study data will be quantitatively synthesised	9
	15b	If data are appropriate for quantitative synthesis, describe planned summary measures, methods of handling data and methods of combining data from studies, including any planned exploration of consistency (such as I^2, Kendall's τ)	N/A
	15c	Describe any proposed additional analyses (such as sensitivity or subgroup analyses, meta-regression)	N/A
	15d	If quantitative synthesis is not appropriate, describe the type of summary planned	12
Meta-bias(es)	16	Specify any planned assessment of meta-bias(es) (such as publication bias across studies, selective reporting within studies)	N/A
Confidence in cumulative evidence	17	Describe how the strength of the body of evidence will be assessed (such as GRADE)	N/A

[*] **N/A = Not applicable**

From: Shamseer L, Moher D, Clarke M, Ghersi D, Liberati A, Petticrew M, Shekelle P, Stewart L, PRISMA-P Group. Preferred reporting items for systematic review and meta-analysis protocols (PRISMA-P) 2015: elaboration and explanation. BMJ. 2015 Jan 2;349(jan02 1):g7647.

For peer review only

Additional file 2: MEDLINE search strategy

1 Sedentary Lifestyle/ (6299)

2 (sedentary or sitting or sedentariness or sedentarism).ti. (5726)

3 ((sedentary or sitting or seated) adj5 (behavio* or lifestyle or life-style)).tw. (6310)

4 ((inactiv* or no exercise or nonexercise or non exercise) adj3 (adult? or men or women or males or females or individuals or people)).tw. (2311)

5 (sedentary adj3 (adult? or men or women or males or females or individuals or people or population?)).tw. (4597)

6 ((sitting or sit or seated or stationary or standing) adj3 (task* or time or bout* or work* or break*)).tw. (4112)

7 low energy expenditure.tw. (133)

8 physical* inactiv*.tw. (5972)

9 (leisure time adj5 (physical* activ* or passive or inactiv*)).tw. (3170)

10 "physical activity level*".tw. (5748)

11 ((sitting or lying) adj2 posture*).tw. (913)

12 (prolong* adj2 (reclin* or sit or sitting or seated)).tw. (473)

13 chair rise?.tw. (288)

14 "sit* less".tw. (568)

15 ((light or low) adj "physical activ*").tw. (1601)

16 ((decrease or reduc* or discourag* or lessen*) adj3 (sit or sitting or stand or standing or physical* inactiv*)).tw. (1259)

17 (time adj5 (computer* or television or tv or video game? or videogame? or gaming or screen or media)).tw. (8281)

18 ((watch* or view*) adj5 (television or tv)).tw. (3937)

19 (play* adj5 (video game? or videogame? or computer game?)).tw. (1191)

20 ((computer* or television or tv or video game? or videogame? or gaming) and (sedentary or physical* activity* or sitting or seated or underactiv* or under activ*)).ti. (313)

21 or/1-20 [sedentary behaviour terms] (46598)

For peer review only

22 Program Evaluation/ (55336)

23 "Outcome and Process Assessment (Health Care)"/ (24581)

24 "Process Assessment (Health Care)"/ (4010)

25 process evaluation*.tw. (2102)

26 program* evaluation*.tw. (3453)

27 or/22-26 [process evaluation] (85436)

28 randomized controlled trial.pt. (451253)

29 controlled clinical trial.pt. (92068)

30 randomized.ab. (350327)

31 placebo.ab. (169658)

32 drug therapy.fs. (1985690)

33 randomly.ab. (243952)

34 trial.ab. (362663)

35 groups.ab. (1525897)

36 or/28-35 (3815378)

37 exp animals/ not humans.sh. (4415536)

38 36 not 37 [RCT filter] (3253766)

39 21 and 27 and 38 [sedentary and process evaluation and RCTs] (362)

For peer review only
